# Effects of scavenger receptors-1 class A stimulation on macrophage morphology and highly modified advanced glycation end product-protein phagocytosis

**DOI:** 10.1038/s41598-018-24325-y

**Published:** 2018-04-12

**Authors:** Shinichi Hamasaki, Takuro Kobori, Yui Yamazaki, Atsuhiro Kitaura, Atsuko Niwa, Takashi Nishinaka, Masahiro Nishibori, Shuji Mori, Shinichi Nakao, Hideo Takahashi

**Affiliations:** 1 Department of Anesthesiology, Kindai University, Faculty of Medicine, 377-2 Ohno-Higashi, Osaka-Sayama, Osaka, 589-8511 Japan; 2 Department of Pharmacology, Kindai University, Faculty of Medicine, 377-2 Ohno-Higashi, Osaka-Sayama, Osaka, 589-8511 Japan; 3 Department of Pharmacology, Okayama University Graduate School of Medicine, Dentistry, and Pharmaceutical Sciences, 2-5-1 Shikata-cho, Okayama, Japan; 4 Department of Pharmacy, Shujitsu University, 1-6-1 Nishikawahara, Okayama, Japan

## Abstract

Advanced glycation end-products (AGEs), which comprise non-enzymatically glycosylated proteins, lipids, and nucleic acid amino groups, play an important role in several diseases and aging processes including angiopathy, renal failure, diabetic complications, and neurodegenerative diseases. Among AGE-associated phenotypes, toxic AGEs, glyceraldehyde-derived AGE-2, and glycolaldehyde-derived AGE-3 are involved in the pathogenesis of diabetic complications. In addition, macrophages are reported to remove extracellular AGEs from tissues via scavenger receptors, leading to the progression of atherosclerosis. In the present study, we found that AGE-2 and AGE-3 enhanced their own endocytic uptake by RAW264.7 mouse macrophage-like cells in a concentration-dependent manner. Furthermore, we demonstrated, for the first time, the morphology of phagocytic macrophages and the endocytosis of AGE particles. The toxic AGEs induced the expression of a scavenger receptor, CD204/scavenger receptors-1 class A (SR-A). Notably, an antibody against CD204 significantly prevented toxic AGE uptake. Moreover, an SR-A antagonistic ligand, fucoidan, also attenuated the AGE-2- and AGE-3-evoked uptake in a concentration-dependent manner. These results indicated that SR-A stimulation, at least in part, plays a role in AGE uptake.

## Introduction

Accommodation of blood aldose sugar levels and various natural antioxidant systems are reported to induce the non-enzymatic formation of endogenous advanced glycation end products (AGEs), although once produced, AGEs eventually accumulate as durable macromolecules^1–4^. In turn, the accumulation of AGEs in long-lived tissue proteins of patients with diabetes induces inflammatory mechanisms in tissues. In particular, the toxic AGE structures glyceraldehyde-derived AGE (AGE-2) and glycolaldehyde-derived AGE (AGE-3) have been demonstrated to play an important role in the pathogenesis of renal failure, arteriosclerosis, angiopathy, and retinopathy in these patients^5^.

Macrophages are capable of ingesting extracellular particles by receptor-mediated endocytosis, fluid-phase endocytosis, and/or phagocytosis^6^. Among AGE receptors, which play a role in the pathology associated with the complications of diabetes and ageing, AGEs can interact with two types of cell surface receptors on macrophages^7^. Scavenger receptors including type I and type II macrophage scavenger receptors, such as class B scavenger receptor (CD36), scavenger receptors-1 class A (SR-A, CD204), and lectin-like oxidized low-density lipoprotein receptor 1 (LOX-1), are predominantly involved in AGE capture, removal, and degradation^8^. In addition, receptor for AGE (RAGE), a member of the immunoglobulin superfamily and a class J scavenger receptor^9,10^, along with toll-like receptor (TLR)-4 constitute other types of AGE receptors that initiate specific cellular signalling events in response to AGE exposure^11^. Although binding of AGE to RAGE is not accompanied by endocytosis, RAGE is involved in the internalisation of AGEs, an essential process for mediating intracellular responses^12^, which in turn leads to cellular activation^11^. Moreover, stimulation of CD204 promotes activation of the TLR4-mediated NF-κB signalling pathway^13^. However, the specific receptor(s) involved in AGE uptake remains unclear.

In the present study, we investigated the uptake of AGEs by macrophages in an attempt to understand the nature of AGE interaction with cell surface AGE-receptors and the localisation thereof. In addition, we assessed the effect of a scavenger receptor ligand, fucoidan, on some actions of AGEs.

## Materials and Methods

### Reagents

AGE-modified bovine serum albumin (BSA) (Sigma-Aldrich, St. Louis, MO, USA) was prepared as previously described^14^. Briefly, each protein was incubated under sterile conditions with glyceraldehyde 3- phosphate (AGE-2) (Sigma-Aldrich) or glycolaldehyde (AGE-3) (Sigma-Aldrich) in 0.2 M phosphate buffer (pH 7.4) at 37 °C for 7 days. BSA was incubated under the same conditions. AGE-BSA and BSA were dialysed for 2 days at 4 °C. The endotoxin concentration of AGE at 100 μg/ml was measured at SRL (Okayama, Japan) as 1.2 pg/ml.

The following pharmacological inhibitors and neutralising antibodies (Abs) were used: fucoidan (1–1,000 μg/ml, F8190, Sigma-Aldrich), neutralising Abs against RAGE (20 μg/ml, AF1179), LOX-1 (20 μg/ml, AF1564), SR-A/CD204 (20 μg/ml, AF1797, all R&D Systems, Minneapolis, MN, USA), CD36 (20 μg/ml, MA5-14112, Thermo Fisher Scientific, Waltham, MA, USA), CD163 (10 μg/ml, GTX54458, GeneTex, Irvine, CA, USA), and CD206 (10 μg/ml, ab8918, Abcam, Cambridge, UK).

### Cell culture

The mouse macrophage cell line RAW264.7 (DS Pharma Biomedical, Osaka, Japan) was grown in Dulbecco’s modified Eagle medium containing 2 mM glutamine and 10% heat-inactivated foetal bovine serum at 37 °C and 5% CO_2_.

### Fluorescent labelling of BSA, AGE-2, and AGE-3 using Alexa Fluor 488 C5 maleimide

BSA, AGE-2, and AGE-3 were fluorescently labelled as described previously with some modification^15,16^. Briefly, each protein was incubated with 20 times the amount of Alexa Fluor 488 C5 maleimide (Thermo Fisher Scientific) at room temperature for 2 h in phosphate buffered saline (PBS) and then dialysed with PBS at 4 °C for 2 days. Total protein concentration was quantified by the Bradford method^17^ using a Bradford protein assay kit (Bio-Rad Laboratories, Kidlington, UK). Alexa Fluor 488-labelled compound fluorescence intensity was measured using ARVO MX 1420 (PerkinElmer Japan, Yokohama, Japan) (excitation: 485 nm, emission: 535 nm). The strengths of Alexa Fluor 488-BSA, -AGE-2, or -AGE-3 per unit dosage were each adjusted by adding respective unlabelled proteins.

### Flow cytometric analysis for macrophage phagocytosis of BSA, AGE-2, and AGE-3

RAW264.7 cells were seeded at 1.0 × 10^5^ cells/well in 24-well plates and incubated with Alexa Fluor 488-BSA, -AGE-2 or, -AGE-3 at concentrations of 0.2–200 μg/ml for 10 min, 1, 2, or 4 h. For experiments using neutralising Abs or pharmacological inhibitors except for fucoidan, cells were pre-treated with neutralising Abs against each receptor for 1 h before incubating with Alexa Fluor 488-BSA, -AGE-2, or -AGE-3 at 200 μg/ml for 1 h. For experiments using fucoidan, cells were concomitantly treated with fluorescently labelled-BSA, -AGE-2, or, -AGE-3 at 200 μg/ml and fucoidan at concentrations of 1–1,000 μg/ml for 1 h. Subsequently, cells were harvested and processed twice by rinsing with FACS wash buffer consisting of PBS supplemented with 2.5% normal horse serum, 0.1% sodium azide, and 10 mM HEPES followed by centrifugation (200 × *g*, 5 min, 4 °C). Subsequently, 300 µl PBS (−) was added to the residue and cells were stained with propidium iodide (PI) (2 µg/ml, Dojindo Laboratories, Kumamoto, Japan) to exclude PI-positive dead cells. Thereafter, analysis was performed using FACS CantoII (BD Biosciences, San Jose, CA, USA) and the data were processed using BD FACSDiva software (BD Biosciences) to determine the mean fluorescence intensity (MFI) of Alexa Fluor 488-labelled AGEs.

### Confocal fluorescence microscopy (CFM) for macrophage phagocytosis of BSA, AGE-2, and AGE-3

RAW264.7 cells were fluorescently labelled with PKH26 (red) (MINI26, Sigma-Aldrich) according to the manufacturer’s protocol. PKH26-labelled RAW264.7 cells seeded at 4.0 × 10^5^ cells/dish in 35-mm glass-bottom dishes (Matsunami Glass, Kishiwada, Japan) were allowed to attach for 1 h followed by treatment with 200 μg/ml Alexa Fluor 488-BSA, -AGE-2, or -AGE-3 for 10 min or 4 h. The fluorescence images of Alexa Fluor 488-AGEs and PKH26-labelled RAW264.7 cells were captured at 0.5–0.8-μm intervals for the *z*-axis at original magnification × 400 using a confocal laser C2 microscope (Nikon, Tokyo, Japan).

In another experiment, RAW264.7 cells seeded at 4.0 × 10^5^ cells/dish in 35-mm glass-bottom dishes were allowed to attach for 1 h followed by treatment with 200 μg/ml Alexa Fluor 488-BSA, -AGE-2, or -AGE-3 for 4 h with or without of fucoidan (500 μg/ml). The fluorescence images of Alexa Fluor 488-AGEs were captured as described above.

### Flow cytometric analysis for surface receptors on RAW264.7 cells

Flow cytometric analysis was performed as described above with some modifications. RAW264.7 cells seeded at 1.0 × 10^5^ cells/well in 24-well plates were incubated with AGEs and BSA (0.2–200 μg/ml) for 1 or 4 h. In some experiments, cells were pre-treated for 1 h with neutralising Ab against CD204 (20 μg/ml) or fucoidan at a concentration of 1–1,000 μg/ml prior to or concomitantly with AGE treatment (200 μg/ml for 1 h). Subsequently, cells were harvested and rinsed with FACS wash buffer followed by centrifugation (200 × *g*, 5 min, 4 °C), then stained with anti-mouse Abs against phycoerythrin (PE)-conjugated CD204 (4 ng, 130-102-328, Miltenyi Biotec, Bergisch Gladbach, Germany), PE-conjugated TLR4 (50 ng, 12-9041-80, Thermo Fisher Scientific), PE-conjugated LOX-1 (0.5 µl, FAB1564P, R&D Systems), allophycocyanin (APC)-conjugated CD36 (25 ng, 102-612, BioLegend, San Diego, CA, USA), APC-conjugated RAGE (50 ng, LS-C212626, LifeSpan BioSciences, Seattle, WA, USA), fluorescein isothiocyanate (FITC)-conjugated CD163 (200 ng, bs-2527R-FITC, Bioss, Woburn, MA, USA), or FITC-conjugated CD206 (200 ng, MCA2235F, Bio-Rad Laboratories, Berkeley, CA, USA) at 4 °C for 30 min. After rinsing with FACS wash buffer followed by centrifugation (200 × *g*, 5 min, 4 °C), 300 µl of PBS (−) was added to the residue followed by staining with PI (2 µg/ml), FACS CantoII analysis, and data processing using BD FACSDiva software to determine the MFI of each surface membrane receptor.

### Confocal laser scanning immunofluorescence microscopy

After adhering RAW264.7 cells (4 × 10^5^ cell/dish) to 35-mm glass-bottom dishes for 1 h, cells were incubated with Alexa Fluor 488-labelled AGEs (200 ng/ml) for 4 h. Then, cells were washed with PBS (−) and fixed with 4% paraformaldehyde (PFA) at room temperature for 30 min followed by washing with PBS-T (PBS pH 7.6 with 0.1% Tween-20). Subsequently, cells were incubated in blocking buffer containing 1% BSA, 0.3 M glycine in PBS-T at room temperature for 1 h to permeabilise the cell membrane and block non-specific protein-protein interactions. Cells were next incubated with normal rabbit IgG (1:100 dilution for negative control) or Ab directed against CD204 (1:100 dilution, PA5-22957, Thermo Fisher Scientific) in blocking buffer at 4 °C overnight. After rinses in PBS-T, cells were incubated with Alexa Fluor 594-conjugated secondary Ab against rabbit IgG (1:500 dilution, A-11037, Thermo Fisher Scientific) at room temperature for 1 h. Then, cells were washed with PBS-T and photomicrographs were taken at 0.5–0.8-μm intervals for the *z*-axis at original magnification × 400 with a confocal laser C2 microscope.

### Transmission electron microscope (TEM) analysis

RAW264.7 cells (8.0 × 10^5^ cells/dish) were attached on 35-mm dishes for 1 h, treated with AGE-2 or AGE-3 (200 μg/ml) for 4 h, rinsed with 0.1 M PBS pH 7.4 at 4 °C for 1 h, and fixed with 2.5% (v/v) glutaraldehyde in 0.1 M PBS pH 7.4 at 4 °C overnight. After rinsing with 0.1 M PBS pH 7.4 at 4 °C for 1 h, cells were post-fixed with 1% (w/v) osmium tetroxide or at 4 °C for 2 h, washed with 0.1 M PBS pH 7.4 at 4 °C for 1 h, and dehydrated in an ascending ethanol series (50, 70, and 80% at 4 °C for 15 min each; 90 and 95% at room temperature for 15 min; 99.5% at room temperature overnight; and 100% for 30 min). The samples were embedded in an epoxy resin at 50 °C for 1 day and 60 °C for 2 days. Ultrathin sections (80 nm) were cut using a microtome, collected on grids, stained with 3% uranyl acetate and lead citrate, and observed using a TEM HT-7700 (Hitachi High-Technologies Corporation, Tokyo, Japan).

### Immunoelectron microscopy

RAW264.7 cells (8.0 × 10^5^ cells) were incubated for 1 h on 35-mm dishes, washed in buffer, and treated with 200 μg/ml BSA, AGE2, or AGE3. Thereafter, cells were rinsed with 0.1 M PBS and fixed with 4% PFA phosphate buffer solution for 10 min. After washing in 20 mM Tris-HCl pH 7.6, the cells were incubated in 1% BSA/10% normal goat serum/0.3 M glycine in PBS-T for 1 h to permeabilise the cells and block non-specific protein-protein interactions. The cells were then incubated with rabbit anti-AGE (BSA-AGE and HSA-AGE) polyclonal Ab (Ab23722, Abcam) overnight at 4 °C. The secondary Ab of labelled polymer combined with peroxidase and anti-rabbit IgG (H + L) (GHP516G, Biocare Medical, Pacheco, CA, USA) was applied for 1 h, and AGE protein localisation visualised with 3, 3′-diaminobenzidine (DAB) chromogen system (Dako, Carpenteria, CA, USA). After rinsing with 0.1 M PBS, cells were post-fixed with 2% (w/v) osmium tetroxide for 1 h, washed with 0.1 M PBS, dehydrated in an ascending ethanol series (50, 70, 80, 90, 95, and 99.5%) for 5 min each and dehydrated in 100% ethanol for 30 min. The samples were embedded in an epoxy resin at 60 °C for 1 day. Ultrathin sections were cut, stained with lead citrate, and observed using a TEM HT-7700.

### Statistical analyses

Data are presented as the means ± SEM. Statistical analyses were performed using Prism version 3 software (GraphPad Software, LaJolla, CA, USA). Statistical significance was assessed using a one-way analysis of variance followed by Dunnett’s or Tukey’s test for multiple comparisons. A value of *p* < 0.05 was considered significant.

## Results

### AGE-BSA uptake by macrophages

Serum AGE levels are elevated over 2-fold in patients with diabetes (approximately 25 μg/ml) and show almost an 8-fold increase in patients with diabetes requiring haemodialysis (approximately 80 μg/ml) when compared with healthy individuals^18,19^. The effect of AGEs at concentrations ranging from 0.1 to 500 μg/ml has been determined by *in vitro* studies using macrophages^20,21^. In the present study, we prepared and used the Alexa Fluor 488 -labelled or unlabelled AGE-2, AGE-3, and BSA at concentrations ranging from 0.2 to 200 μg/ml, none of which affected the viability of RAW264.7 macrophage cells at 24 h (see Supplementary Fig. S1).

**Figure 1:**
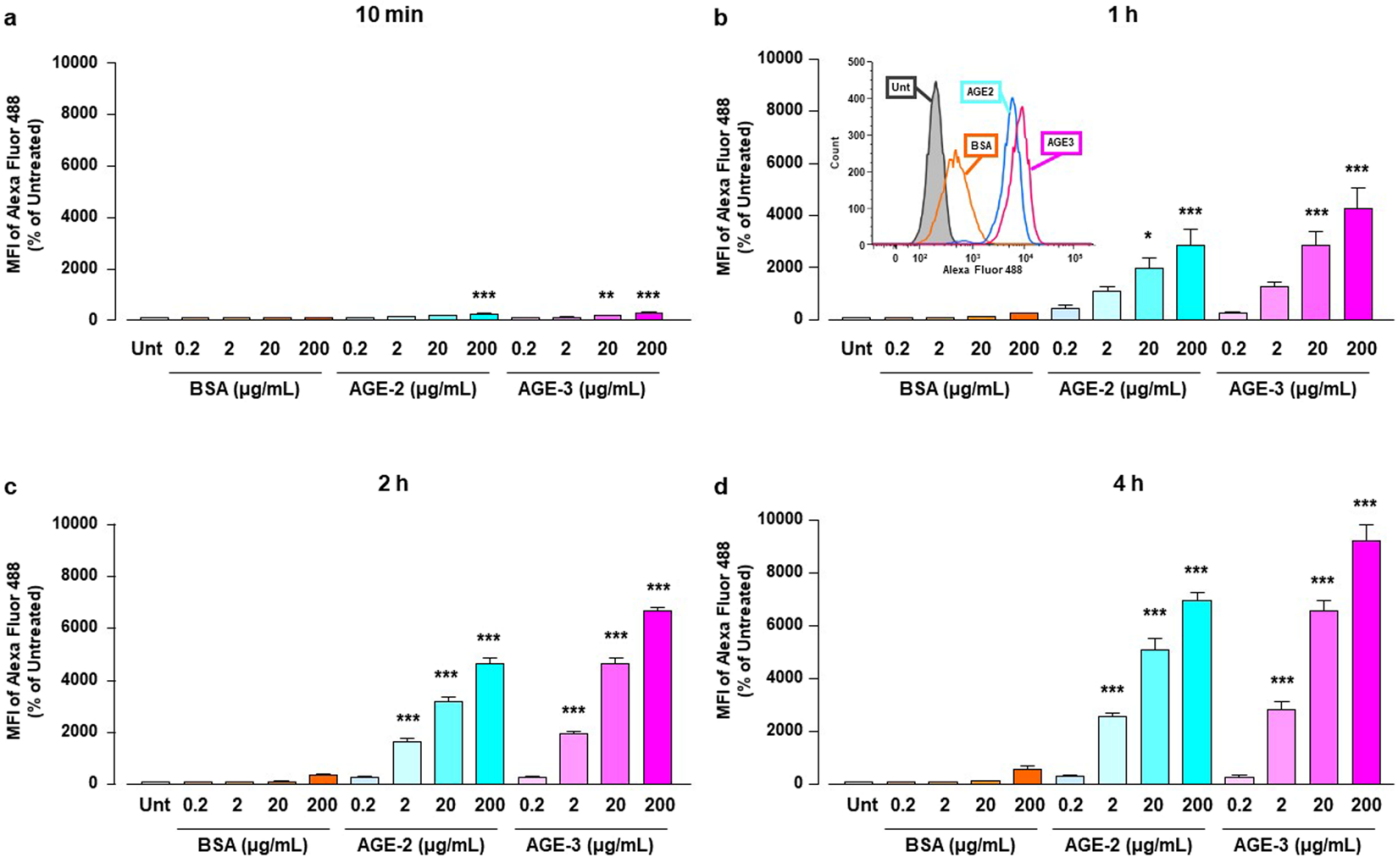
Endocytic AGEs uptake in cultured RAW264.7 cells. BSA, AGE-2, and AGE-3 were fluorescently labelled with Alexa Fluor 488. RAW264.7 cells seeded at 4 × 10^5^ cells were incubated with fluorescent BSA, AGE-2, or AGE-3 at concentrations ranging from 0.2 to 200 μg/ml for the indicated periods: 10 min (**a**), 1 h (**b**), 2 h (**c**), or 4 h (**d**). Cellular uptakes of fluorescent BSA, AGE-2, and AGE-3 were determined by flow cytometry (n = 3). Each column represents the MFIs of Alexa Fluor 488-labelled AGEs relative to medium only (Unt, Untreated) after 10 min incubation, which was arbitrarily defined as 100%. Data are presented as the means ± SEM and were analysed using a one-way ANOVA followed by Tukey’s test. ^***^p < 0.001, ^**^p < 0.01, ^*^p < 0.05 compared with the value for Unt. The insert in (b) shows an overlay of a representative monoparametric histogram of CD204 expression in RAW264.7 cells incubated for 1 h with medium only (Untreated; greyscale), 200 μg/ml BSA (orange line), AGE-2 (blue line), or AGE-3 (red line).

As shown in Fig. 1, we examined time and dose-dependent changes in the uptake of fluorescently labelled-AGE-2, AGE-3, and BSA by RAW264.7 macrophage cells from 10 min to 4 h after treatment, by means of quantitative flow cytometry. Treatment with AGE-2 and AGE-3 at 200 μg/ml significantly enhanced their uptake by cells beginning at 10 min and persisting up to 4 h. Similarly, the uptake of AGEs incubated at 20 μg/ml was significantly and continuously increased from 1 to 4 h. AGE-2 and AGE-3 at 2 μg/ml also significantly enhanced their own uptake from 2 h to 4 h. In contrast, treatment with AGEs at 0.2 μg/ml and BSA at any concentration had no effect on their own uptake.

**Figure 2:**
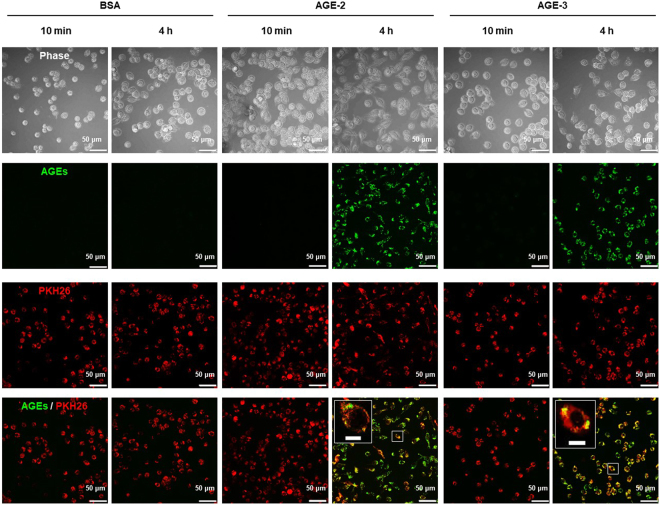
Confocal laser scanning immunofluorescence microscopy for AGE uptake. Prior to the experiments, RAW264.7 cells were fluorescently labelled using PKH26 (red) and seeded at 4 × 10^5^ cells/well followed by culturing for 10 min and 4 h with 200 μg/ml BSA, AGE-2, or AGE-3, all of which were fluorescently labelled with Alexa Fluor 488 (green). The incorporated fluorescent BSA, AGE-2, and AGE-3 in cells was determined by confocal fluorescence microscopy. Each panel of BSA, AGE-2, or AGE-3 indicates, as follows; (upper) phase contrast, (upper middle) Alexa Fluor 488-labelled AGEs, (lower middle) PKH26-labelled cell membrane, or (lower) merge of Alexa Fluor 488 and PKH26. All micrographs in this figure were taken at the same magnification (×400). Scale bar indicates 50 μm. The clipped images at the upper left of the merge panels in AGE-2 or AGE-3 show higher magnification images of the white rectangle region in the corresponding panel. Scale bar indicates 10 μm.

**Figure 3:**
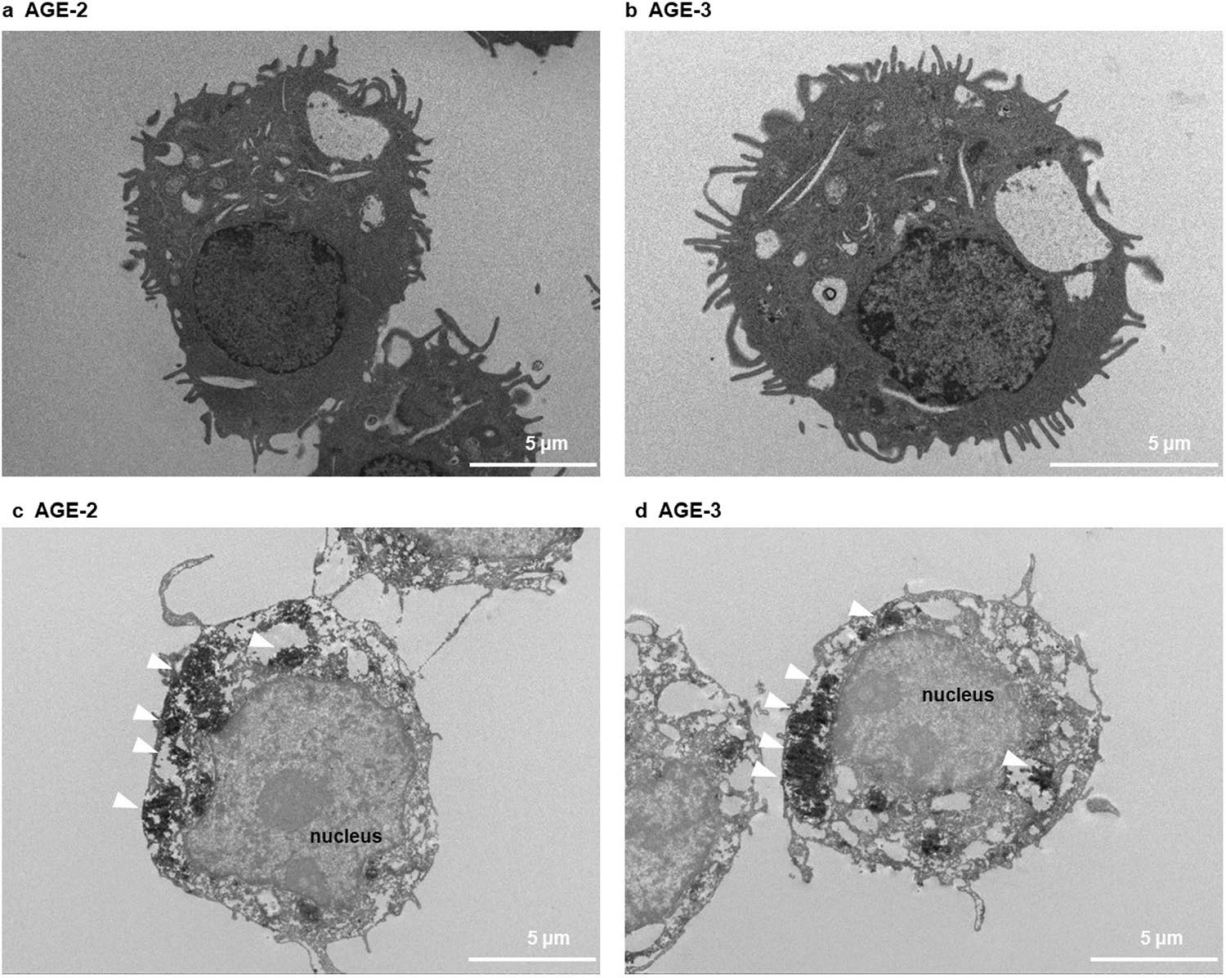
Effects of AGE-2 and AGE-3 on RAW 264.7 cell morphology and phagocytosis. Macrophages seeded at 8 × 10^5^ cells/well were cultured for 4 h with AGE-2 or AGE-3 at 200 μg/ml. Transmission electron microscopy (TEM) indicated the morphological change of RAW264.7 cells treated by AGE-2 (**a**) or AGE-3 (**b**). TEM in combination with immunohistochemical analysis showed intracellular black particles, which reflect the incorporation of AGE-2 (**c**) or AGE-3 (**d**), both of which react with the anti-AGE Ab post-stained with a diaminobenzidine labelling procedure. All the micrographs in this figure were taken at the same magnification (see scale bar). The arrows in panel (**c**) or (**d**) show intracellular particles of AGE-2 or AGE-3 within a phagosome, respectively.

In the present study, the endocytic uptake of AGEs by RAW264.7 cells was confirmed, for the first time, by means of CFM (Fig. 2), time-lapse live cell imaging (see Supplementary Videos S1, S2, and Fig. S2), in addition to TEM analysis (Fig. 3 and Supplementary Fig. S3). The CFM data showed that after incubation of PKH26-labelled RAW264.7 cells with Alexa Fluor 488-labelled AGE-2 or AGE-3, the accumulation of AGE particles (green) not only near the plasma membrane but also in the cytoplasm of cells (red) at 4 h were apparently facilitated in comparison with those at 10 min, implying an enhancement in the internalisation of AGEs into the cellular compartment (Fig. 2). As is the case in live-cell imaging (Supplementary Videos S1, S2, and Fig. S2), AGE-2 and AGE-3 changed the morphology of RAW264.7 cells, with the morphology of phagocytic cells appearing to have an elongated fibroblast-like shape, extending their pseudopodia or end-feet to AGEs, resulting in AGE engulfment. The cytoplasm at the cell ends was thin and elongated to produce a tapered appearance, and almost all cells exhibited only a few pseudopodia. Notably, the elongated cells had numerous cytoplasmic vacuolations, suggesting a phagocyte morphological appearance.

TEM analysis revealed that incubation with AGE-2 or AGE-3 for 4 h greatly induced the morphological changes in RAW264.7 cells, in which plasma membrane fusion at the rims of the engulfing cup might lead the closure of the phagosome (Fig. 3a and b). Moreover, by means of immunoelectron microscopy analysis to observe endocytic uptake of AGEs, we found that AGE-2 and AGE-3 were ingested by RAW264.7 cells and trapped in phagosomes, as each particle was surrounded by a membrane vesicle in the cytoplasmic region at 4 h after incubation (Fig. 3c and d).

### Expression of scavenger receptors in macrophages

**Figure 4:**
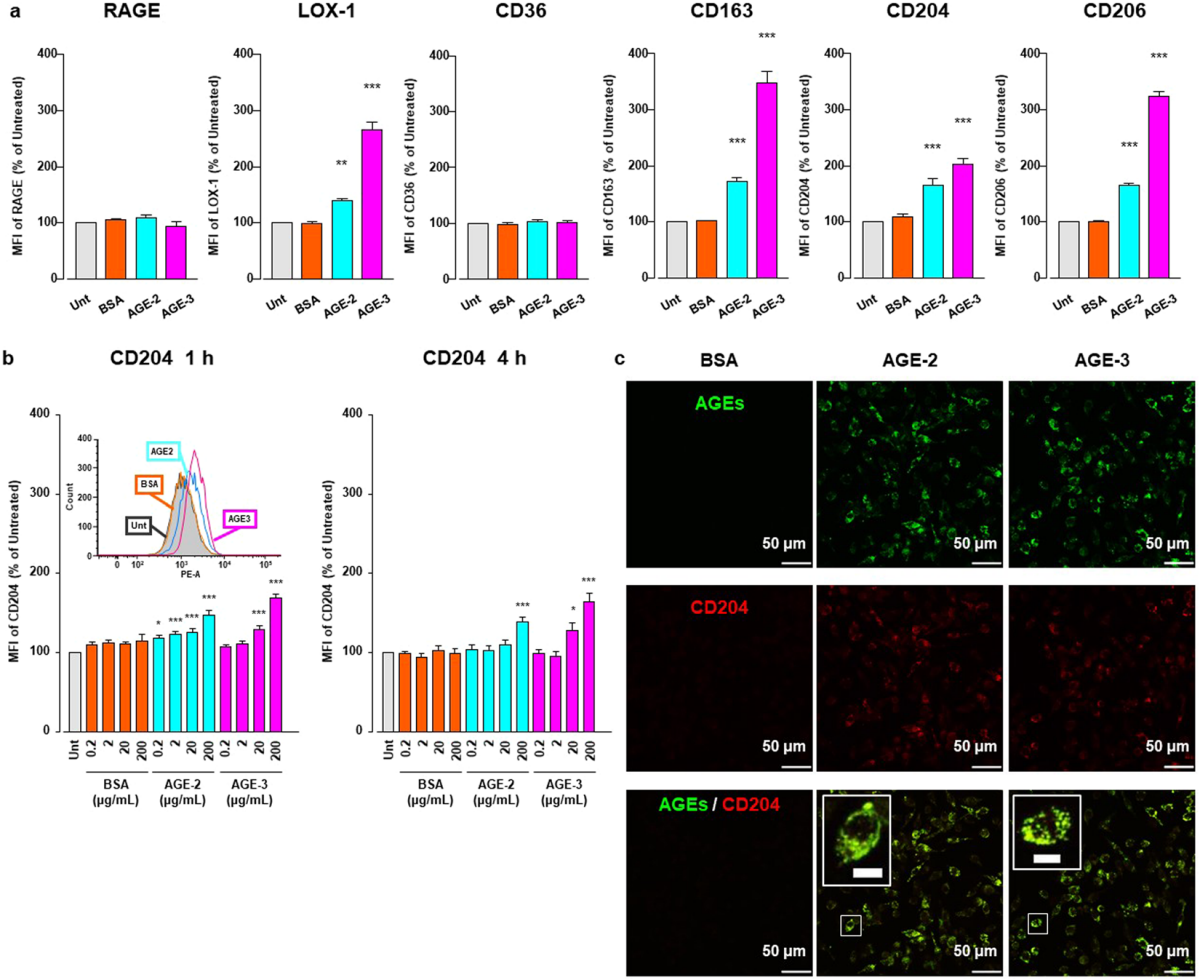
AGE-2 and AGE-3 effects on scavenger receptor expression. (**a**) RAW264.7 cells seeded at 2 × 10^5^ per well incubated for 1 h with 200 μg/ml AGE-2, AGE-3, or BSA were stained with specific FITC-labelled Abs against CD163 or CD206, specific PE-labelled CD204 or LOX-1, and APC-labelled CD36 or RAGE, and relative mean fluorescence intensities (MFIs) determined by flow cytometry (n = 6). Each column represents respective surface protein MFIs relative to medium only (Untreated, Unt) after 1 h incubation, arbitrarily defined as 100%. Data are presented as the means ± SEM and analysed using one-way ANOVA followed by Tukey’s test. ^***^p < 0.001, ^**^p < 0.01 compared with the Unt value. (**b**) For RAW264.7 cells seeded at 2 × 10^5^ per well incubated with increasing concentrations AGE-2, AGE-3, and BSA (0.2–200 μg/ml) for 1 or 4 h, relative CD204 MFIs were determined by flow cytometry (n = 4–6). Each column represents MFIs relative to Unt after 1 or 4 h incubation, arbitrarily defined as 100%. Data are presented as the means ± SEM and analysed using one-way ANOVA followed by Tukey’s test. ^***^p < 0.001, ^*^p < 0.05 compared with Unt. Insert in 1 h shows overlay of representative monoparametric histogram of CD204 expression in RAW264.7 cells incubated with medium only (Untreated; greyscale), 200 μg/ml BSA (orange), AGE-2 (blue), or AGE-3 (red line). (**c**) RAW264.7 cells seeded at 4 × 10^5^ per well were cultured for 4 h with 200 μg/ml Alexa Fluor 488 (green)-labelled BSA, AGE-2, or AGE-3. CD204 intracellular localisation was visualized using anti-CD204 Ab and Alexa Fluor 594 (red) labelled secondary Ab, with confocal fluorescence microscopy. Rabbit IgG served as an anti-CD204 Ab isotype-matched control. All micrographs are shown at the same magnification (×400). Scale bar indicates 50 μm. Each BSA, AGE-2, or AGE-3 panel indicates, as follows; (upper) Alexa Fluor 488-labelled AGEs, (middle) Alexa Fluor 594-labelled CD204, or (lower) merged image. Clipped images at the upper left of the merged panel in AGE-2 or AGE-3 represent higher magnifications of the white rectangle region in the corresponding panel. Scale bar indicates 10 μm.

Because RAGE, SR-A, and LOX-1 have been identified as receptors for toxic AGEs^5^, we asked whether treatment of RAW264.7 cells with AGE-2, AGE-3, and BSA at 200 μg/ml for 1 h might influence the expression of these toxic AGE receptors in addition to CD36, haemoglobin scavenger receptor (CD163), and mannose receptor-1 (CD206), all of which are also known as receptors for AGEs^22^. We found that the toxic AGEs significantly enhanced the surface expression of CD163, CD204, CD206, and LOX-1, but not CD36 and RAGE in RAW264.7 cells (Fig. 4a). Moreover, an isotype-matched control of anti-CD204 Ab detected no obvious changes in the fluorescence signal between each treatment group (see Supplementary Fig. S4a) and Alexa Fluor 488-labelled AGE-2 and AGE-3 at 200 μg/ml also evoked the expression of CD204 on RAW264.7 cells (see Supplementary Fig. S4b). In the absence of AGEs, the expression of scavenger receptors showed no marked change.

Next, we investigated time-and dose-dependent effects of AGEs and BSA incubation for 1 or 4 h at concentrations ranging from 0.2 to 200 μg/ml on the surface expression of CD204 in RAW264.7 cells by flow cytometry. AGE-2 and AGE-3 enhanced the expression of CD204 at 1 and 4 h in a concentration-dependent manner (Fig. 4b). When we assumed that the effects of AGE-2 and AGE-3 on uptake and CD204 expression were maximal at the concentration of 200 μg/ml at 1 h after incubation, the EC50 values of AGE-2 and AGE-3 for these two functions were calculated to be approximately 20 and 30 μg/ml, respectively. Thus, increases in the uptake of AGEs and surface CD204 expression in RAW264.7 cells were induced by AGEs at a similar concentration range.

It is generally accepted that different features impose a temporal progression of phagosome formation according to the following sequence of events: (A) particle surface molecules are engaged by phagocyte receptors, after which actin-driven membrane dynamics facilitate the detection of surrounding particles. (B) Engagement and activation of the receptor induces signalling cascades, which leads to actin reorganisation. (C) Actin polymerisation progresses around the particle accompanied by further engagement of receptors, which in turn activates actin clearance and focal exocytosis at the base of the cup, thereby facilitating particle engulfment. (D) Once the particle is fully surrounded, membrane fusion at the rims of the cup seals the phagosome and separates it from the plasma membrane^23^. By means of CFM, we examined the localisation of CD204/SR-A in RAW264.7 cells after a 4-h incubation with Alexa Fluor 488-labelled AGEs and BSA. Notably, SR-A positive staining (red) appeared to be concentrated not only at the cell surface plasma membrane but also at intracellular endosomes of RAW264.7 cells (Fig. 4c). Furthermore, phagocytosed AGEs (green) were highly co-localised with SR-A/CD204 (red), implying that the uptake of AGEs might be, at least, accompanied by engagement of CD204 possibly operating as a key receptor for AGEs.

### Involvement of SR-A stimulation in AGE uptake

Although the stimulation of CD36 and SR-A is involved in AGE uptake by macrophages^24–26^, CD204 also plays a pivotal role in the endocytic uptake of AGE by macrophages^27^. Thus, it remains unclear which scavenger receptors are actually involved in toxic AGE uptake.

**Figure 5:**
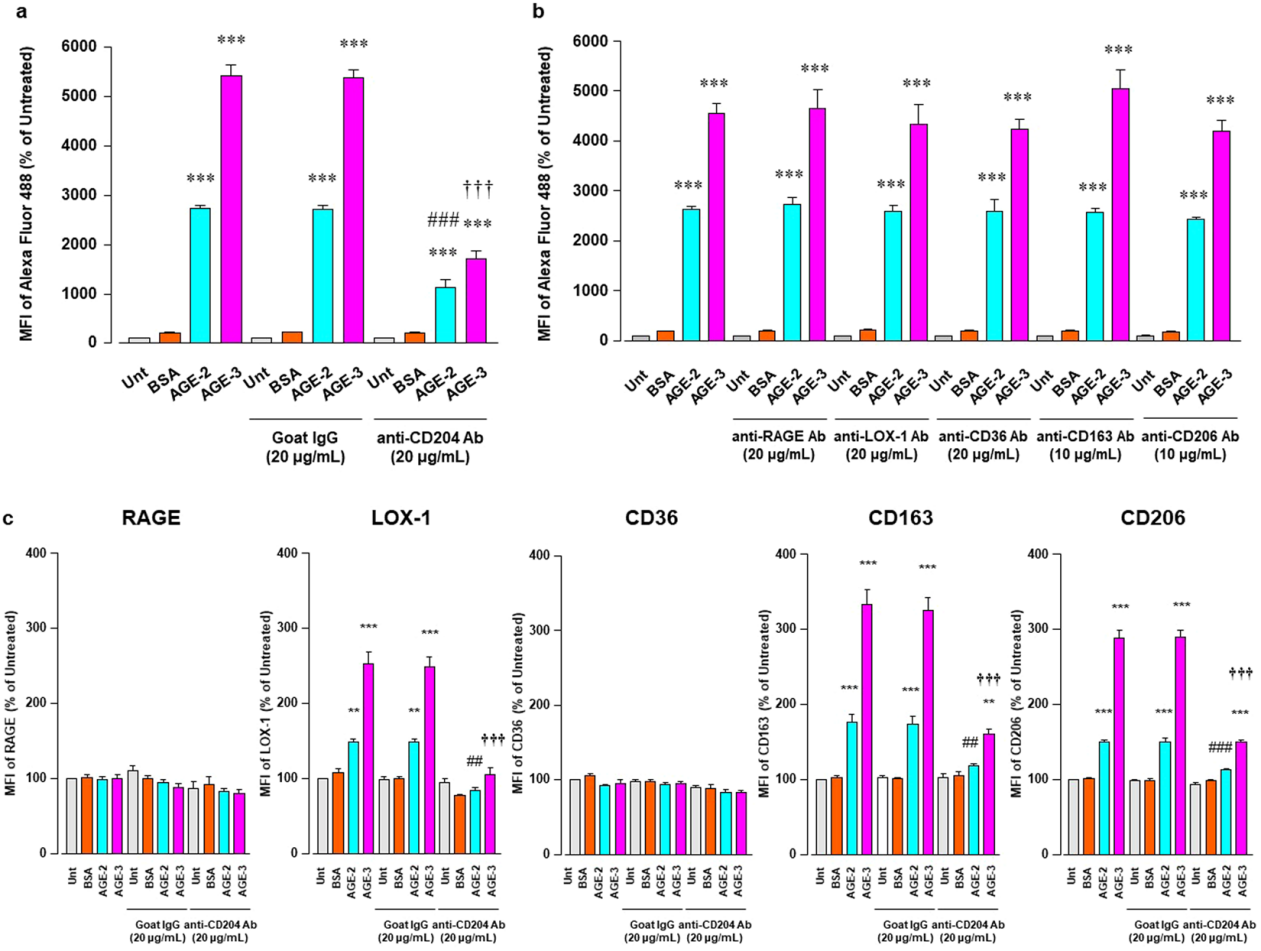
Inhibitory effect of Abs against scavenger receptors for AGE uptake RAW264.7 cells seeded at 2 × 10^5^ per well were pre-incubated with or without neutralizing Abs against scavenger receptors at 20 or 10 μg/ml for 1 h followed by culturing with BSA, AGE-2, or AGE-3 at 200 μg/ml for 1 h. (**a**) Neutralizing effect of anti-CD204 Ab on the uptake of fluorescent AGE-2, AGE-3, and BSA was determined by flow cytometry (n = 3). Goat IgG was used as an isotype-matched control of anti-CD204 Ab. (**b**) Neutralizing effect of each Ab against RAGE, LOX-1, CD36, CD163, or CD206 on the uptake of fluorescent BSA, AGE-2, or AGE-3 was determined by flow cytometry (n = 3–10). (**c**) Effect of anti-CD204 Ab on the expression of RAGE, LOX-1, CD36, CD163, and CD206 was determined by flow cytometry (n = 4–5). Each column represents the MFIs of the respective surface membrane proteins relative to the medium only group (Unt, Untreated) after 1 h incubation, which was arbitrarily defined as 100%. Data are presented as the means ± SEM and were analysed using one-way ANOVA followed by Tukey’s test. ^***^p < 0.001, ^**^p < 0.01 compared with the value for Unt. ^###^*p* < 0.001, ^##^*p* < 0.01 compared with the value for AGE-2 alone. ^†††^*p* < 0.001 compared with the value for AGE-3 alone.

To confirm which scavenger receptors are responsible for AGE-initiated phagocytosis, we observed changes in the uptake of Alexa Fluor 488-labelled AGEs and BSA under the condition where each receptor was blocked by neutralising Abs against RAGE, LOX-1, CD36, CD163, CD204, or CD206. We found that anti-CD204 Ab at 20 μg/ml inhibited the effect of AGE-2 and AGE-3 at 200 μg/ml on their uptake enhancement by RAW264.7 cells (Fig. 5a), whereas neutralising Abs against other receptors had no effect (Fig. 5b). Moreover, anti-CD204 Ab at 20 μg/ml prevented increases in the expression of LOX-1, CD204, and CD206 by treatment with AGE-2 and AGE-3, but had no influence on that of RAGE and CD36 (Fig. 5c). These results indicated that increases in the AGE uptake capacity of RAW264.7 cells may primarily be due to the activation of SR-A.

### Antagonistic effect of fucoidan on AGE actions

**Figure 6:**
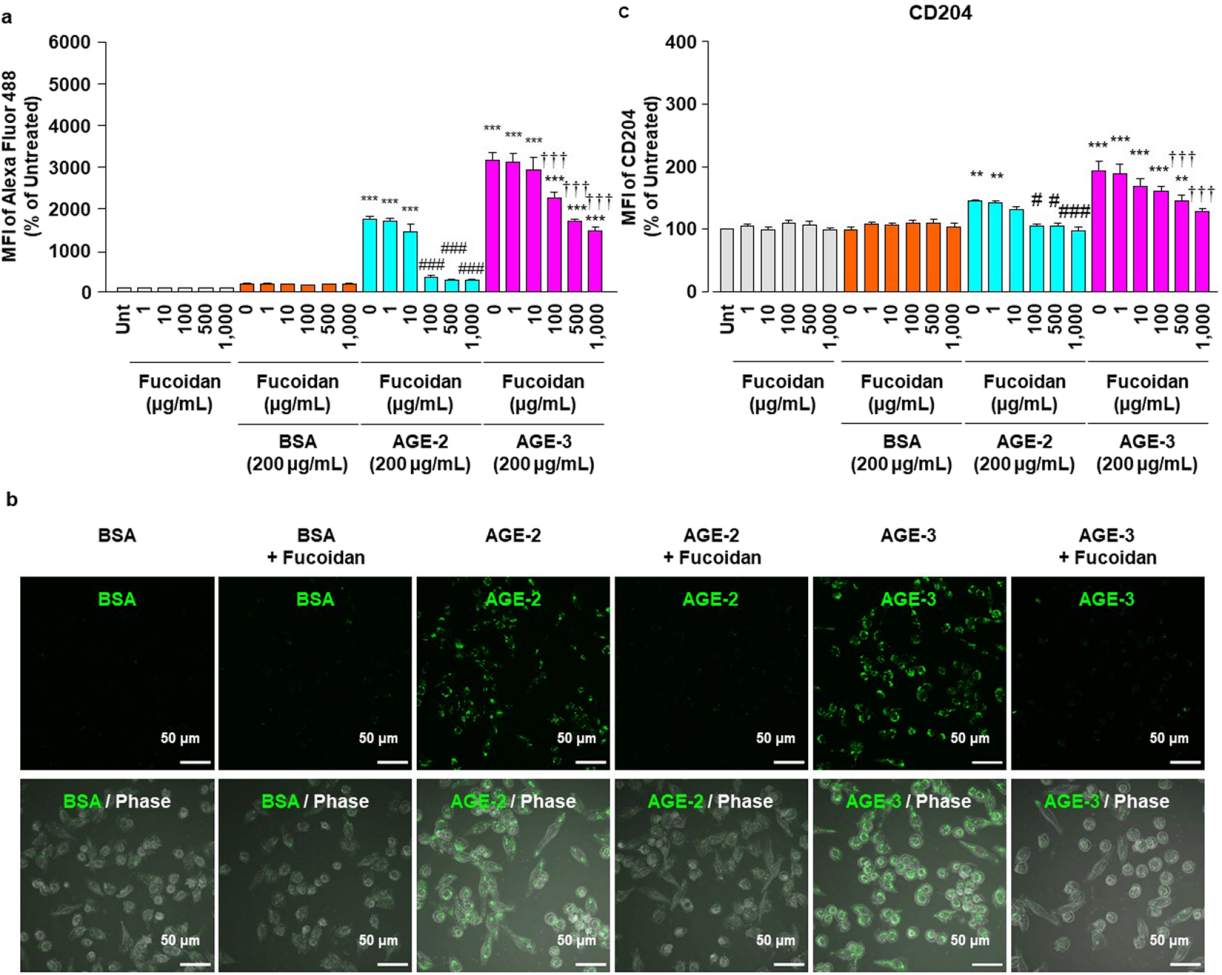
Effects of fucoidan on AGE actions. RAW264.7 cells seeded at 2 × 10^5^ per well were concomitantly treated with fucoidan at increasing concentrations from 1 to 1000 μg/ml in the presence and absence of BSA, AGE-2, or AGE-3 at 200 μg/ml for 1 h. (**a**) Cellular uptakes of fluorescent BSA, AGE-2, or AGE-3 were determined by flow cytometry (n = 3). (**b**) Representative confocal laser scanning immunofluorescence images of fluorescent BSA, AGE-2, or AGE-3 (green) and of each overlay with phase contrast after incubating RAW264.7 cells for 4 h with Alexa Fluor 488-labelled BSA, AGE-2, or AGE-3 in the presence or absence of fucoidan (500 μg/ml) administered concomitantly with AGE. All the micrographs were taken at the same magnification (×400). Scale bar indicates 50 μm. (**c**) The relative MFIs of CD204 expression were determined by flow cytometry (n = 9). Each column represents the MFI of CD204 relative to the medium only group (Unt, Untreated) after 1 h incubation, which was arbitrarily defined as 100%. Data are presented as the means ± SEM and were analysed using one-way ANOVA followed by Tukey’s test. ^***^p < 0.001, ^**^p < 0.01 compared with the value for Unt. ^###^*p* < 0.001, ^#^*p* < 0.05 compared with the value for AGE-2 alone. ^†††^*p* < 0.001 compared with the value for AGE-3 alone.

Previous study using RAW264.7 cells and other types of macrophages indicated that SR-A-mediated phagocytosis of pathogens is specifically inhibited by the SR-A ligand, fucoidan^28–30^. However, it remains unknown whether fucoidan influences macrophage phagocytosis of AGEs. Therefore, we finally asked whether fucoidan at concentrations ranging from 1 to 1000 μg/ml, which is almost the same as that in recent studies^31–33^, had any influence on the AGE uptake capacity of RAW264.7 cells and the surface expression of CD204 as determined by flow cytometry at 1 h after incubation with AGEs. The enhancements in the AGE-2 and AGE-3 uptake capacity of RAW264.7 cells in association with an increase in the surface CD204 expression were attenuated by concomitant treatment with fucoidan in a concentration-dependent manner (Fig. 6). In particular, fucoidan used at 500 and 1000 μg/ml markedly inhibited increases in the AGE uptake capacity and the surface expression of CD204 (Fig. 6). When we assumed that an inhibitory effect of fucoidan on these responses elicited by AGE-2 or AGE-3 were maximal at 1000 μg/ml, the IC50 values were calculated to be approximately 40 and 20 μg/ml for AGE uptake and CD204 expression, respectively.

## Discussion

Nagai *et al*. reported that highly modified but not mildly modified AGE-BSA is recognised by scavenger receptors such as SR-A, CD36, and SR-BI on the cell surface membrane of macrophages^34^. A growing body of evidence suggests that, among these scavenger receptors, SR-A plays a key role in the endocytic uptake of AGE proteins by macrophages or macrophage-derived cells^27^. Although AGE-3 undergoes endocytosis with subsequent lysosomal degradation in RAW264.7 cells, suggesting the presence of a high affinity binding site for glycolaldehyde^35^, the involvement of scavenger receptors in toxic AGE uptake remains to be understood. In the present study, we found that AGE-2 as well as AGE-3, which comprise supra-physiological highly modified AGE-BSA, enhanced their own endocytic uptake by RAW264.7 cells in a concentration-dependent manner (Fig. 1). Furthermore, the alteration in cell morphology induced by AGE-2 and AGE-3 was associated with modulation of uptake, as assessed by the mean number of particles reflecting internalised AGEs within RAW264.7 cells (Figs 2 and 3). The toxic AGEs also increased the expression of SR-A/CD204 (Fig. 4). AGE-elicited uptake was attenuated by blockade of CD204 using an anti-CD204 Ab (Fig. 5a) but not by several Abs including those raised against RAGE, LOX-1, CD36, CD163, or CD206 (Fig. 5b). In addition, an SR-A inhibitor, fucoidan, dramatically inhibited the facilitation of AGE-induced self-uptake and the surface CD204 expression, possibly by acting as an antagonist for SR-A (Fig. 6a and b). Based on these results, we suggested that the recognition of AGEs by SR-A/CD204, at least, plays a pivotal role in AGE-2- and AGE-3-induced endocytic uptake. However, because we have used a supra-physiological highly modified protein in our experiments, future work should investigate the uptake of mildly modified AGE-proteins as well.

To characterise possible differences between receptor-independent fluid-phase endocytosis (pinocytosis) and receptor-mediated endocytosis of AGEs, we investigated the effect of a pinocytosis inhibitor, 5-ethyl- N-isopropyl amiloride (EIPA)^36^, on AGE uptake (see Supplementary Fig. S8). EIPA moderately reduced the uptake levels of AGE-2 and AGE-3 to 70–80%, whereas it had no significant influence on the increase in the surface expression of CD204, indicating that the fluid phase endocytosis was partially involved in the AGE uptake as part of the compensatory mechanisms for removing AGE.

Identification of signalling pathways may lead to better understanding of the mechanism by which macrophages uptake AGEs. Although it is reported that SR-A does not contain an intracellular activating signal sequence, SR-A interacts with other signalling and transport proteins to mediate intracellular signalling events^37^. Previous studies using RAW264.7 cells indicated that lipopolysaccharide (LPS) stimulates TLR-4 to augment phagocytosis^38^ and to induce NF-*κ*B activation^39^. SR-A acts as an enhancer for NF-*κ*B activation through the TLR-4 induced by LPS stimulation in macrophages^40^. However, it remains unknown whether TLR-4 stimulation and NF-*κ*B signalling are involved in AGE uptake by RAW264.7 cells. In the present study, we confirmed that AGE-2 and AGE-3 enhanced the expression of TLR-4 through the stimulation of SR-A (see Supplementary Fig. S5), whereas the AGE-induced uptake capacity and expression of CD204 were independent of TLR-4 stimulation (see Supplementary Fig. S6). In addition, AGE treatment induced almost no changes in NF-*κ*B activation (see Supplementary Figs S7a and S7b), and enhancement in the surface expression of CD204 by AGE treatment was independent of NF-*κ*B activation (see Supplementary Fig. S7c), implying that the NF-*κ*B pathway has little influence on AGE phagocytosis by RAW264.7 macrophages. Moreover, the surface expression level of CD204 in RAW264.7 cells was poorly enhanced by LPS stimulation (data not shown). These observations raise the possibility that the receptor and signalling pathway responsible for the uptake of AGEs and LPS may vary depending on the type of stimuli. Further study to understand the mechanism underlying uptake of AGEs by macrophages should therefore be performed.

In response to rising AGE levels, the endogenous clearance system effected by scavenger receptors maintains normal AGE homeostasis at the sites of AGE deposits; however, this system is disrupted under the conditions where AGE levels are chronically elevated in diabetes mellitus and with age^35,41,42^. AGE accumulation is detected in macrophage-derived foam cells at the early stage and vascular smooth muscle cell-derived foam cells at the advanced stage in human atherosclerotic lesions^43^. Moreover, the recognition, endocytosis, and degradation of AGEs lead to foam-cell formation induced by macrophage accumulation, resulting in arteriosclerosis^44^. In addition, SR-A in macrophages plays critical roles in the progression of foam cell formation and atherosclerosis^45^, indicating that extracellular AGEs in the sub-endothelial space of arterial walls are endocytosed by macrophages through stimulation of SR-A.

Fucoidan, a fucose-containing sulphated polysaccharide derived from brown seaweeds, affects variable pathophysiological processes including inflammation, vascular physiology, carcinogenesis, and oxidative stress^46,47^. Fucoidan significantly attenuates atherosclerotic plaque formation and enhances plaque stability by decreasing serum lipids and inhibiting macrophage infiltration^48^. It is reported that fucoidans of different molecular weight exert differential effects on the viability and function of immune cells^49^. Although high molecular weight (HMW) fucoidan constitutes the main fraction of fucoidan responsible for its immuno-stimulatory activity, HMW fucoidans exhibit less activity related to anti-coagulant and pro-apoptotic properties than low molecular weight (LMW) fucoidans. In the present study, we used the HMW fucoidan extracted from *Fucus vesiculosus*. Although the details of *F. vesiculosus* fucoidan uptake, tissue distribution, and final metabolic fate are not well understood, the distribution of fucoidan is suggested to occur following its oral uptake^50^. Moreover, fucoidan has been investigated as an anti-oxidant, anti-cancer, and anti-inflammatory agent^51,52^. Therefore, the present study indicated SR-A as constituting a possible a therapeutic target in the complications of diabetes mellitus and the aging process.

In conclusion, the present study demonstrated that the modulation of RAW264.7 cell morphology in response to AGE-2 and AGE-3, both of which are ligands for SR-A, leads to activation of macrophages with concomitant enhancement in AGE phagocytosis capacity. Additional studies are required to determine the significance of these observations occurring in the context of complications in diabetes and aging. Investigation of the optimising interaction between AGEs and macrophages in tissues of an *in vivo* model might further reveal the mechanism by which diabetes mellitus accelerates inflammation and might provide an attractive approach for effective treatment of complications in this disease.

## Electronic supplementary material

Supplementary Information

Video S1

Video S2

## References

[1] Vlassara H, Palace MR (2002). Diabetes and advanced glycation endproducts. J. Intern. Med..

[2] Stern D, Yan SD, Yan SF, Schmidt AM (2002). Receptor for advanced glycation endproducts: a multiligand receptor magnifying cell stress in diverse pathologic settings. Adv. Drug Deliv. Rev..

[3] Schmidt AM (1994). Receptor for advanced glycation end products (AGEs) has a central role in vessel wall interactions and gene activation in response to circulating AGE proteins. Proc. Natl. Acad. Sci. USA.

[4] Schaper NC, Havekes B (2012). Diabetes: impaired damage control. Diabetologia.

[5] Takeuchi M, Takino J, Yamagishi S (2010). Involvement of the toxic AGEs (TAGE)-RAGE system in the pathogenesis of diabetic vascular complications: a novel therapeutic strategy. Curr. Drug Targets.

[6] Guest CB (2007). Phagocytosis of cholesteryl ester is amplified in diabetic mouse macrophages and is largely mediated by CD36 and SR-A. PLoS One.

[7] Byun K (2017). Advanced glycation end-products produced systemically and by macrophages: A common contributor to inflammation and degenerative diseases. Pharmacol. Ther..

[8] Smedsrød B, Melkko J, Araki N, Sano H, Horiuchi S (1997). Advanced glycation end products are eliminated by scavenger-receptor-mediated endocytosis in hepatic sinusoidal Kupffer and endothelial cells. Biochem. J..

[9] PrabhuDas MR (2017). A consensus definitive classification of scavenger receptors and their roles in health and disease. J. Immunol..

[10] He M (2011). Receptor for advanced glycation end products binds to phosphatidylserine and assists in the clearance of apoptotic cells. EMBO Rep..

[11] Neeper M (1992). Cloning and expression of a cell surface receptor for advanced glycosylation end products of proteins. J. Biol. Chem..

[12] Sevillano N (2009). Internalization of the receptor for advanced glycation end products (RAGE) is required to mediate intracellular responses. J. Biochem..

[13] Seimon TA, Obstfeld A, Moore KJ, Golenbock DT, Tabas I (2006). Combinatorial pattern recognition receptor signaling alters the balance of life and death in macrophages. Proc. Natl. Acad. Sci. USA.

[14] Takeuchi M (2000). Immunological evidence that non-carboxymethyllysine advanced glycation end-products are produced from short chain sugars and dicarbonyl compounds *in vivo*. Mol. Med..

[15] Miki Y (2014). Macrophage recognition of toxic advanced glycosylation end products through the macrophage surface-receptor nucleolin. Biol. Pharm. Bull..

[16] Miki Y (2015). Nucleolin is a receptor for maleylated-bovine serum albumin on macrophages. Biol. Pharm. Bull..

[17] Bradford MM (1976). A rapid and sensitive method for the quantitation of microgram quantities of protein utilizing the principle of protein-dye binding. Anal. Biochem..

[18] Yamagishi S (1997). Advanced glycation end products-driven angiogenesis *in vit*ro. Induction of the growth and tube formation of human microvascular endothelial cells through autocrine vascular endothelial growth factor. J. Biol. Chem..

[19] Makita Z, Vlassara H, Cerami A, Bucala R (1992). Immunochemical detection of advanced glycosylation end products *in vivo*. J. Biol. Chem..

[20] Takahashi HK (2009). Advanced glycation end products subspecies-selectively induce adhesion molecule expression and cytokine production in human peripheral blood mononuclear cells. J. Pharmacol. Exp. Ther..

[21] Jin X (2015). Advanced glycation end products enhance macrophages polarization into M1 phenotype through activating RAGE/NF-kappaB pathway. Biomed. Res. Int..

[22] Horiuchi S, Sakamoto Y, Sakai M (2003). Scavenger receptors for oxidized and glycated proteins. Amino Acids.

[23] Jaumouillé, V. & Grinstein, S. Molecular mechanisms of phagosome formation. *Microbiol. Spectr*. **4** (2016). 10.1128/microbiolspec.MCHD-0013-2015 27337463

[24] Stifano G, Christmann RB (2016). Macrophage involvement in systemic sclerosis: Do we need more evidence?. Curr. Rheumatol. Rep..

[25] Mantovani A, Biswas SK, Galdiero MR, Sica A, Locati M (2013). Macrophage plasticity and polarization in tissue repair and remodelling. J. Pathol..

[26] Ott C (2014). Role of advanced glycation end products in cellular signaling. Redox Biol..

[27] Sano H, Nagai R, Matsumoto K, Horiuchi S (1999). Receptors for proteins modified by advanced glycation endproducts (AGE)–their functional role in atherosclerosis. Mech. Ageing Dev..

[28] Wang R, Chandawarkar RY (2010). Phagocytosis of fungal agents and yeast via macrophage cell surface scavenger receptors. J. Surg. Res..

[29] Thelen T (2010). The class A scavenger receptor, macrophage receptor with collagenous structure, is the major phagocytic receptor for Clostridium sordellii expressed by human decidual macrophages. J. Immunol..

[30] O’Brien DK, Melville SB (2003). Multiple effects on Clostridium perfringens binding, uptake and trafficking to lysosomes by inhibitors of macrophage phagocytosis receptors. Microbiology.

[31] Li H (2017). Fucoidan from Fucus vesiculosus suppresses hepatitis B virus replication by enhancing extracellular signal-regulated Kinase activation. Virol. J..

[32] Jiang X, Yu J, Ma Z, Zhang H, Xie F (2015). Effects of fucoidan on insulin stimulation and pancreatic protection via the cAMP signaling pathway *in vivo* and *in vitro*. Mol. Med. Rep..

[33] Wang Y (2015). Fucoidan exerts protective effects against diabetic nephropathy related to spontaneous diabetes through the NF-kappaB signaling pathway *in vivo* and *in vitro*. Int. J. Mol. Med..

[34] Nagai R (2007). The ligand activity of AGE-proteins to scavenger receptors is dependent on their rate of modification by AGEs. Biochim. Biophys. Acta.

[35] Nagai R (2000). Glycolaldehyde, a reactive intermediate for advanced glycation end products, plays an important role in the generation of an active ligand for the macrophage scavenger receptor. Diabetes.

[36] Ghigo E (2008). Ameobal pathogen mimivirus infects macrophages through phagocytosis. PLoS Pathog..

[37] Kelley JL, Ozment TR, Li C, Schweitzer JB, Williams DL (2014). Scavenger receptor-A (CD204): a two-edged sword in health and disease. Crit. Rev. Immunol..

[38] Sigola LB, Fuentes AL, Millis LM, Vapenik J, Murira A (2016). Effects of Toll-like receptor ligands on RAW 264.7 macrophage morphology and zymosan phagocytosis. Tissue Cell.

[39] Shim DW (2015). Anti-Inflammatory action of an antimicrobial model peptide that suppresses the TRIF-dependent signaling pathway via inhibition of Toll-like receptor 4 endocytosis in lipopolysaccharide-stimulated macrophages. PLoS One.

[40] Yu H (2012). Scavenger receptor A (SR-A) is required for LPS-induced TLR4 mediated NF-kappaB activation in macrophages. Biochim. Biophys. Acta.

[41] Vlassara H, Moldawer L, Chan B (1989). Macrophage/monocyte receptor for nonenzymatically glycosylated protein is upregulated by cachectin/tumor necrosis factor. J. Clin. Invest..

[42] Vlassara H, Uribarri J, Cai W, Striker G (2008). Advanced glycation end product homeostasis: exogenous oxidants and innate defenses. Ann. N. Y. Acad. Sci..

[43] Kume S (1995). Immunohistochemical and ultrastructural detection of advanced glycation end products in atherosclerotic lesions of human aorta with a novel specific monoclonal antibody. Am. J. Pathol..

[44] Nagai R, Fujiwara Y, Mera K, Otagiri M (2007). Investigation of pathways of advanced glycation end-products accumulation in macrophages. Mol. Nutr. Food Res..

[45] Yu XH, Fu YC, Zhang DW, Yin K, Tang CK (2013). Foam cells in atherosclerosis. Clin. Chim. Acta.

[46] Fitton JH (2011). Therapies from fucoidan; multifunctional marine polymers. Mar. Drugs.

[47] Pomin VH (2012). Fucanomics and galactanomics: current status in drug discovery, mechanisms of action and role of the well-defined structures. Biochim. Biophys. Acta.

[48] Yokota T, Nomura K, Nagashima M, Kamimura N (2016). Fucoidan alleviates high-fat diet-induced dyslipidemia and atherosclerosis in ApoE(shl) mice deficient in apolipoprotein E expression. J. Nutr. Biochem..

[49] Jang JY, Moon SY, Joo HG (2014). Differential effects of fucoidans with low and high molecular weight on the viability and function of spleen cells. Food Chem. Toxicol..

[50] Nakazato K, Takada H, Iha M, Nagamine T (2010). Attenuation of N-nitrosodiethylamine-induced liver fibrosis by high-molecular-weight fucoidan derived from Cladosiphon okamuranus. J. Gastroenterol. Hepatol..

[51] Myers SP (2011). A combined Phase I and II open-label study on the immunomodulatory effects of seaweed extract nutrient complex. Biologics.

[52] Li C (2011). Fucoidan, a sulfated polysaccharide from brown algae, against myocardial ischemia-reperfusion injury in rats via regulating the inflammation response. Food Chem. Toxicol..

