# Supplementary material for: Effects of scavenger receptors-1 class A stimulation on macrophage morphology and highly modified advanced glycation end product-protein phagocytosis
Source: Sci Rep. 2018 Apr 12;8:5901. doi: 10.1038/s41598-018-24325-y (PMC5897562; doi:10.1038/s41598-018-24325-y)
Supplement: Supplementary file 1 — Supplementary Information [file 41598_2018_24325_MOESM1_ESM.pdf]

Supplementary Information

Effects of scavenger receptors-1 class A stimulation on macrophage morphology and highly modified advanced glycation end product-protein phagocytosis

Shinichi Hamasaki[1], Takuro Kobori[2], Yui Yamazaki[2], Atsuhiro Kitaura[1], Atsuko Niwa[2], Takashi Nishinaka[2], Masahiro Nishibori[3], Shuji Mori[4], Shinichi Nakao[1] & Hideo Takahashi[2,*]

[1]Department of Anesthesiology, and [2]Department of Pharmacology, Kindai University, Faculty of Medicine, 377-2 Ohno-Higashi, Osaka-Sayama, Osaka, 589-8511, Japan. [3]Department of Pharmacology, Okayama University Graduate School of Medicine, Dentistry, and Pharmaceutical Sciences, 2-5-1 Shikata-cho, Okayama, Japan. [4]Department of Pharmacy, Shujitsu University, 1-6-1 Nishikawahara, Okayama, Japan.

Correspondence and requests for materials should be addressed to H. T., Department of Pharmacology, Faculty of Medicine, Kindai University, 377-2 Ohno-Higashi, Osaka-Sayama, Osaka, 589-8511, Japan. Telephone: +(81)-72-366-0211, Fax: +(81)-72-366-0206. (email: hkt@med.kindai.ac.jp)

Supplementary Figure S1. Influence of AGE treatment on the viability of RAW264.7 cells. RAW264.7 cells were seeded at 1.0 × 10^4 per well in 24-well plates and stimulated by non-labelled AGEs or Alexa Fluor 488-labelled-AGEs at different concentrations ranging from 0.2 to 200 μg/ml. After incubating for 4 h, the percentage of propidium iodide (PI)-positive dead cells per total cells in each treatment group was determined by flow cytometry analysis. None of the non-labelled or fluorescent AGEs showed obvious influence on the viability of RAW264.7 cells at any concentration when compared with the medium only group (Untreated cells; Unt). Data are expressed as the means ± SEM. n = 3. There were no significant differences ($p > 0.05$).

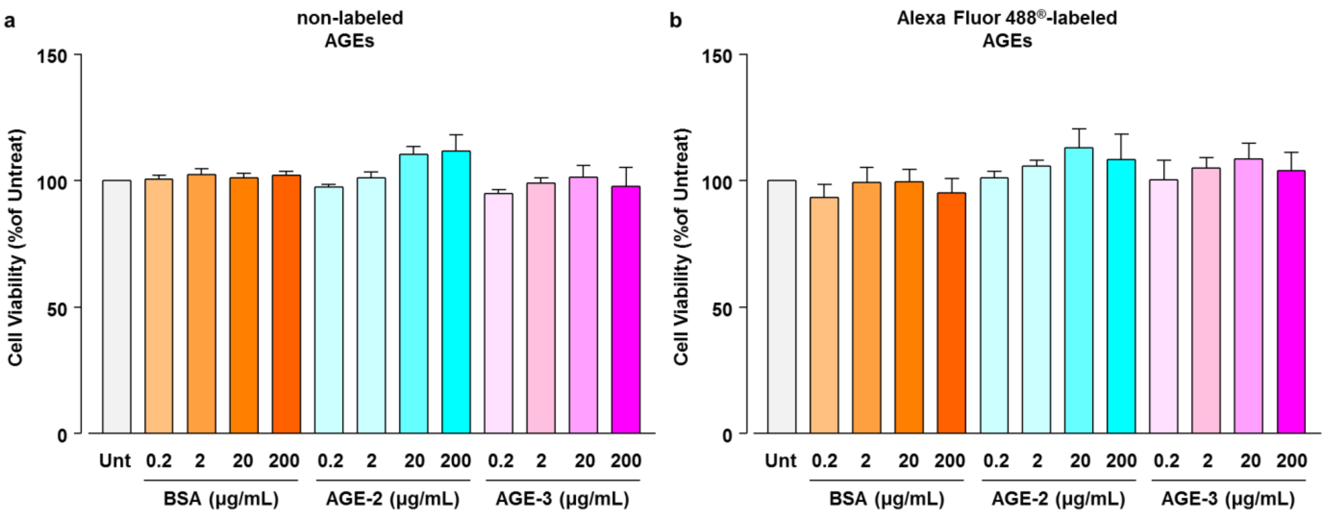

Supplementary Fig. S1

Supplementary Figure S2. Time-lapse live-cell imaging during the endocytic uptake of AGEs. Time-lapse live-cell imaging was performed in the phagocytosis assay in which PKH26 (red)-labelled RAW264.7 cells seeded at 4.0 × 10^5 per dish were incubated in the presence of Alexa Fluor 488-labelled AGE-2 (Supplementary Video S1) or AGE-3 (Supplementary Video S2) at 200 μg/ml for 4 h at 37°C under 5% CO_2. Time-lapse *Z*-stack fluorescence images were overlaid on the phase-contrast images. The time elapsed after starting the movie is indicated in hours:minutes in the bottom right of each panel. (a) Representative series of time-lapse images at 1 h intervals from 0 to 4 h extracted from Supplementary Video S1. Scale bar represents 50 μm. (b) Representative series of time-lapse images at 1 h intervals from 0 to 4 h extracted from Supplementary Video S2. Scale bar represents 50 μm.

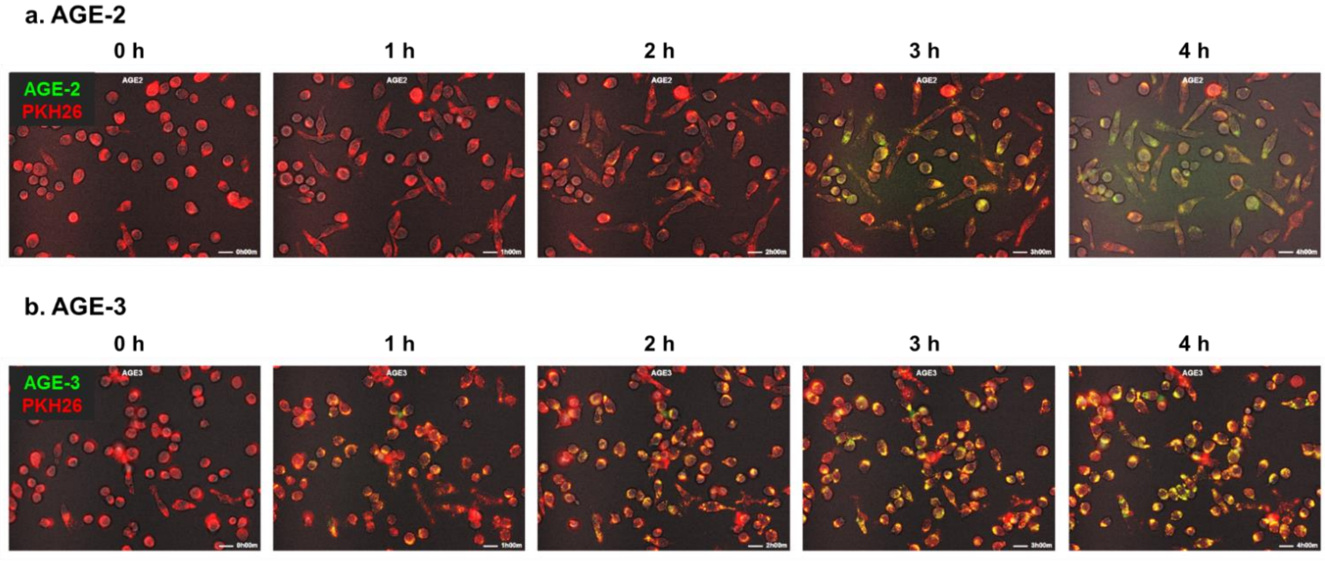

Supplementary Fig. S2

Supplementary Figure S3. Anti-AGE Ab but not its isotype-matched control Ab specifically recognizes intracellular AGEs taken up by RAW264.7 cells. After culturing RAW264.7 cells seeded at 4.0 × 10^5 per dish with 200 μg/ml AGEs for 4 h, cell were reacted with primary Ab against AGE or its isotype-matched control Ab (rabbit IgG), and subsequently stained with Alexa Fluor 488-conjugated anti-rabbit secondary Ab. The endocytic uptake of BSA, AGE-2, or AGE-3 by RAW264.7 cells after 4 h incubation was confirmed by confocal laser scanning immunofluorescence microscopy. The anti-AGE Ab apparently reacted with AGE-2 and AGE-3 but not with BSA, whereas rabbit IgG as a negative control appeared to hardly recognize AGE-2 or AGE-3. Representative fluorescence images of Alexa Fluor 488 (green) (upper panels) and overlay images of phase contrast and Alexa Fluor 488 (green) (lower panels) obtained from each combination of AGE treatment and Abs. Scale bar represents 50 μm.

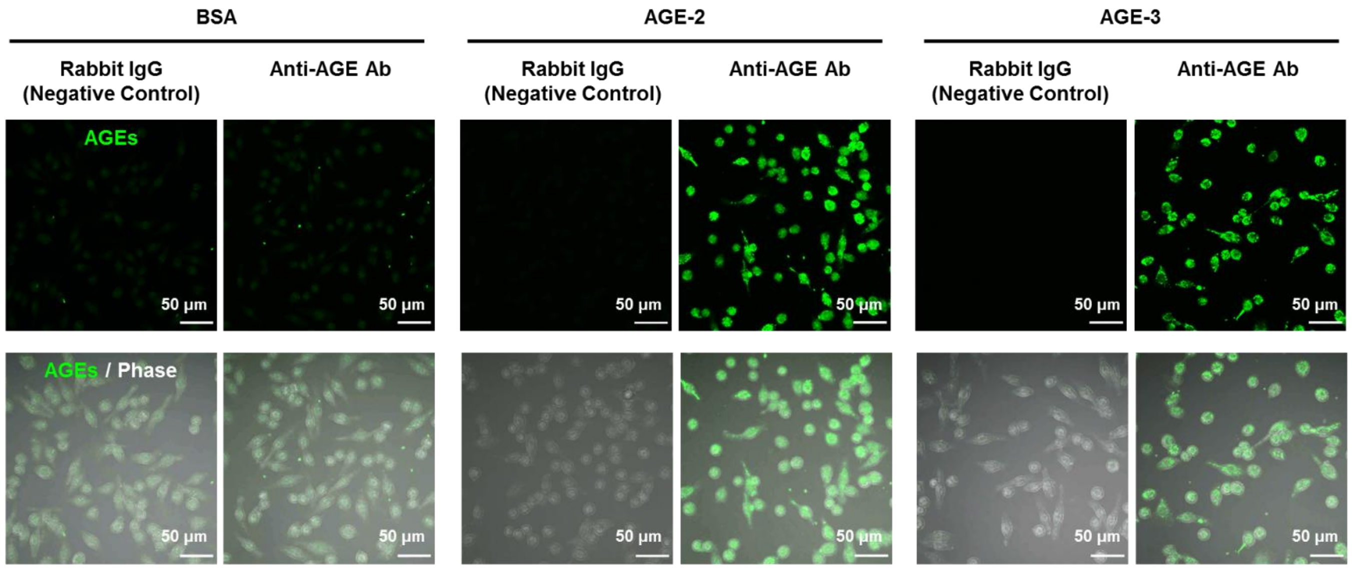

Supplementary Fig. S3

Supplementary Fig. S4. Effect of fluorescent AGEs on the surface expression level of CD204 in RAW264.7 cells. (a) After culturing RAW264.7 cells seeded at 1.0 × 10^5 per well with unlabelled or Alexa Fluor 488-labelled AGEs at 200 μg/ml for 1 h, the surface expression level of CD204 in RAW264.7 cells was measured by flow cytometry analysis. Similar to the effect of unlabelled AGEs, fluorescent AGE-2 and AGE-3 both dramatically increased CD204 expression when compared with the untreated group. Representative monoparametric histogram of CD204 expression in RAW264.7 cells incubated for 1 h with medium only (Untreated, Unt; greyscale), or 200 μg/ml BSA (orange line), AGE-2 (blue line), or AGE-3 (red line). (b, c) After culturing RAW264.7 cells seeded at 1.0 × 10^5 cells with (b) AGE-2 or (c) AGE-3 200 μg/ml for 1 h, cells were incubated with R-phycoerythrin (PE)-conjugated anti-CD204 Ab or its isotype matched control Ab followed by flow cytometry analysis. In RAW264.7 cells treated with AGE-2 or AGE-3, the PE-conjugated anti-CD204 Ab showed apparently high fluorescence signal when compared with its isotype matched control Ab. (b) Representative monoparametric histogram of PE fluorescence intensity in RAW264.7 cells incubated for 1 h with medium only (Untreated, Unt) – isotype matched control Ab (Greyscale), AGE-2 200 μg/mL – isotype matched control Ab (blue dotted line), or AGE-2 200 μg/mL – anti-CD204 Ab (blue line). (c) Representative monoparametric histogram of PE fluorescence intensity in RAW264.7 cells incubated for 1 h with medium only (Untreated, Unt) – isotype matched control Ab (Greyscale), AGE-3 200 μg/mL – isotype matched control Ab (red dotted line), or AGE-3 200 μg/mL – anti-CD204 Ab (red line).

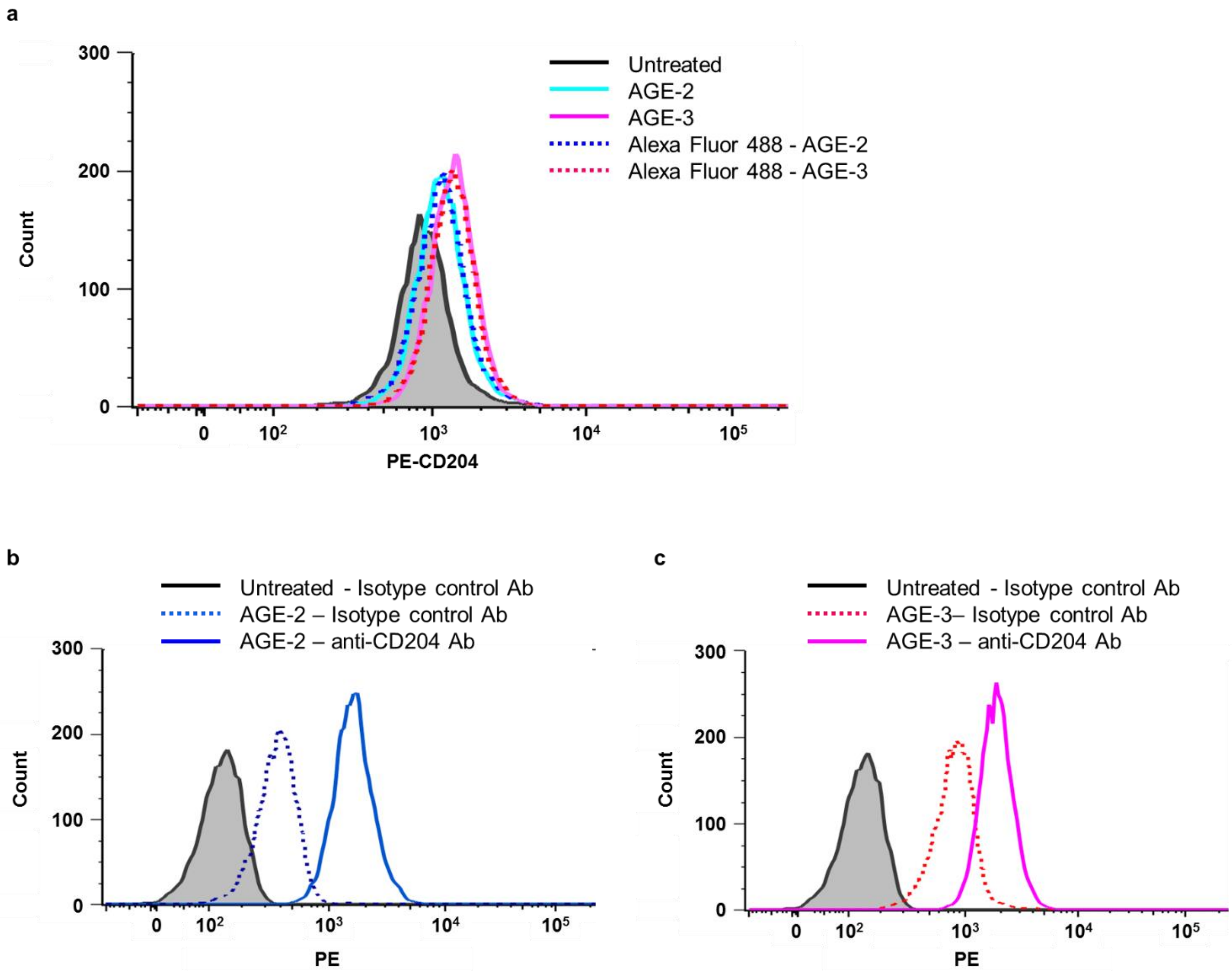

Supplementary Fig. S4

Supplementary Figure S5. AGE-2 and AGE-3 induce the expression of TLR-4 via SR-A. (a) RAW264.7 cells seeded at 1.0 × 10^5 per well were incubated with AGEs at 200 μg/ml for the indicated period as follows; 0 h (upper left), 1 h (upper right), 2 h (lower left), or 4 h (lower right). After AGE treatment, the surface expression levels of TLR-4 in RAW264.7 cells were determined by flow cytometry analysis. Each column represents the MFIs of TLR-4 relative to the medium only group (Untreated; Unt), which was arbitrarily defined as 100 % (n = 6). Data are presented as the means ± SEM and analysed using one-way ANOVA followed by Tukey's test. $^{***}p < 0.001$, $^{*}p < 0.05$ compared with Unt. (b) RAW264.7 cells seeded at 1.0 × 10^5 per well were pre-incubated with 20 μg/ml neutralizing Ab against CD204 or its isotype matched control Ab (Goat IgG) for 1 h followed by treatment with AGEs at 200 μg/ml for 1 h. Subsequently, the surface expression level of TLR-4 in RAW264.7 cells was determined by flow cytometry analysis. AGE-2 and AGE-3 increased TLR-4 expression moderately or significantly, respectively. These effects were suppressed by pre-treatment with an anti-CD204 Ab. Each column represents the MFIs of TLR-4 relative to Unt, which was arbitrarily defined as 100% (n = 4). Data are presented as the means ± SEM and analysed using one-way ANOVA followed by Tukey's test. $^{***}p < 0.001$ compared with the value for Unt. $^{###}p < 0.001$, $^{#}p < 0.05$ compared with the value for AGE-2 alone. $^{†††}p < 0.001$ compared with the value for AGE-3 alone.

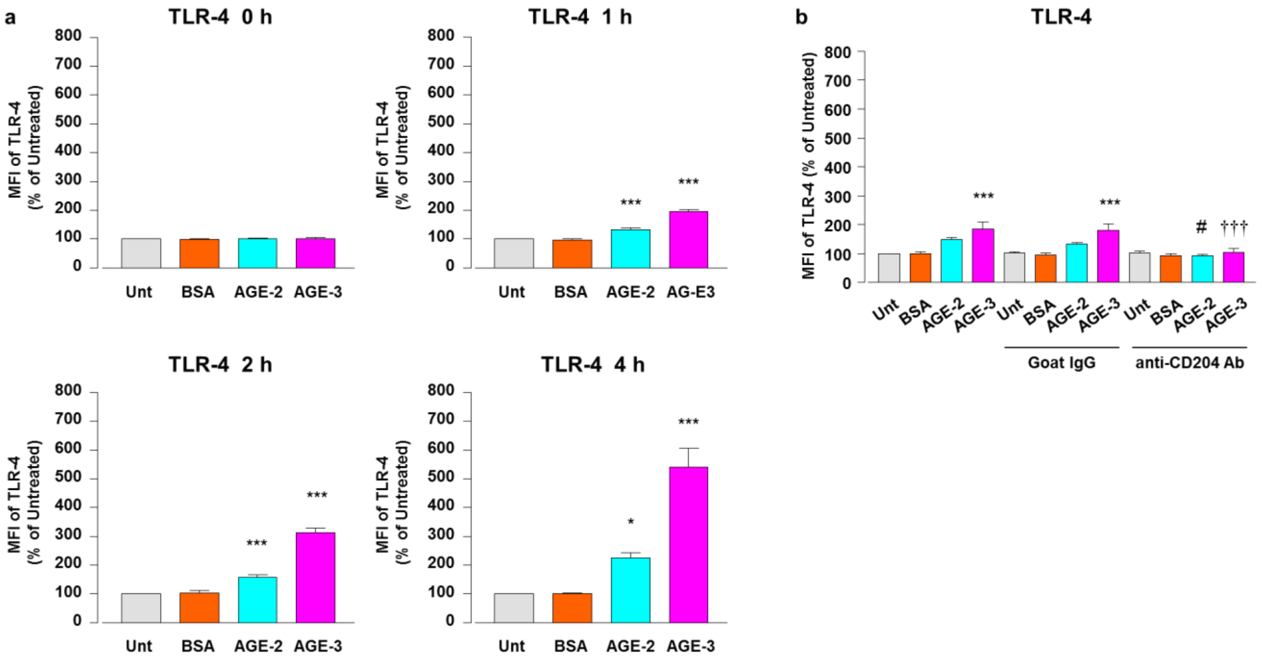

Supplementary Fig. S5

Supplementary Figure S6. Independency of TLR-4 with respect to AGE actions. (a) RAW264.7 cells were incubated with small double-stranded interfering RNAs (siRNAs) (10 pmol/well) for 24 h, after which the cell culture medium was replaced by fresh media. The expression levels of TLR-4 in the whole cellular fraction or the surface membrane were measured by western blotting or flow cytometry analysis, respectively, either immediately or 24 h after replacement of media. The TLR-4 levels in the knock-down cells were reduced by approximately 50-60 % in comparison with that in wild-type cells with effects sustained for 24 h after cessation of the siRNA. For western blotting, each upper column represents the TLR-4 expression level relative to medium only group (Untreated; Unt), which was arbitrarily defined as 100 % (n = 6) and lower panels are typical and full-length images of TLR-4 and glyceraldehyde-3-phosphate dehydrogenase (GAPDH). For flow cytometry, each column represents the MFIs of TLR-4 relative to Unt, which was arbitrarily defined as 100 % (n = 3). (b-e) Effect of a TLR-4 knock down and a TLR-4 antagonist, lipopolysaccharide from *Rhodobacter sphaeroides* (LPS-RS), on the levels of surface CD204 expression and the fluorescent AGEs uptake were analysed by flow cytometry. RAW264.7 cells and those pre-treated with control siRNA, TLR-4 siRNA for 24 h or LPS-RS at 20 μg/ml for 1 h, were incubated for 1 h with 200 μg/ml (b, d) non-fluorescent AGEs or (c, e) Alexa Fluor 488-labelled AGEs. Subsequently, (b, d) the surface expression of CD204 and (c, e) the intracellular uptake of fluorescent AGEs were measured by flow cytometry analysis. AGE-induced increases in the surface CD204 expression and AGEs uptakes were similar between the wild-type, knock-down cells, and the cells pre-treated with LPS-RS. Each column represents (b, d) the MFIs of CD204 relative to Unt or (c, e) those of Alexa Fluor 488-labelled AGEs relative to Unt, which was arbitrarily defined as 100 % (n = 3-4). All data are presented as the means

± SEM and analysed using one-way ANOVA followed by Tukey's test. $^{***}p < 0.001$, $^{*}p < 0.05$ compared with the value for Unt.

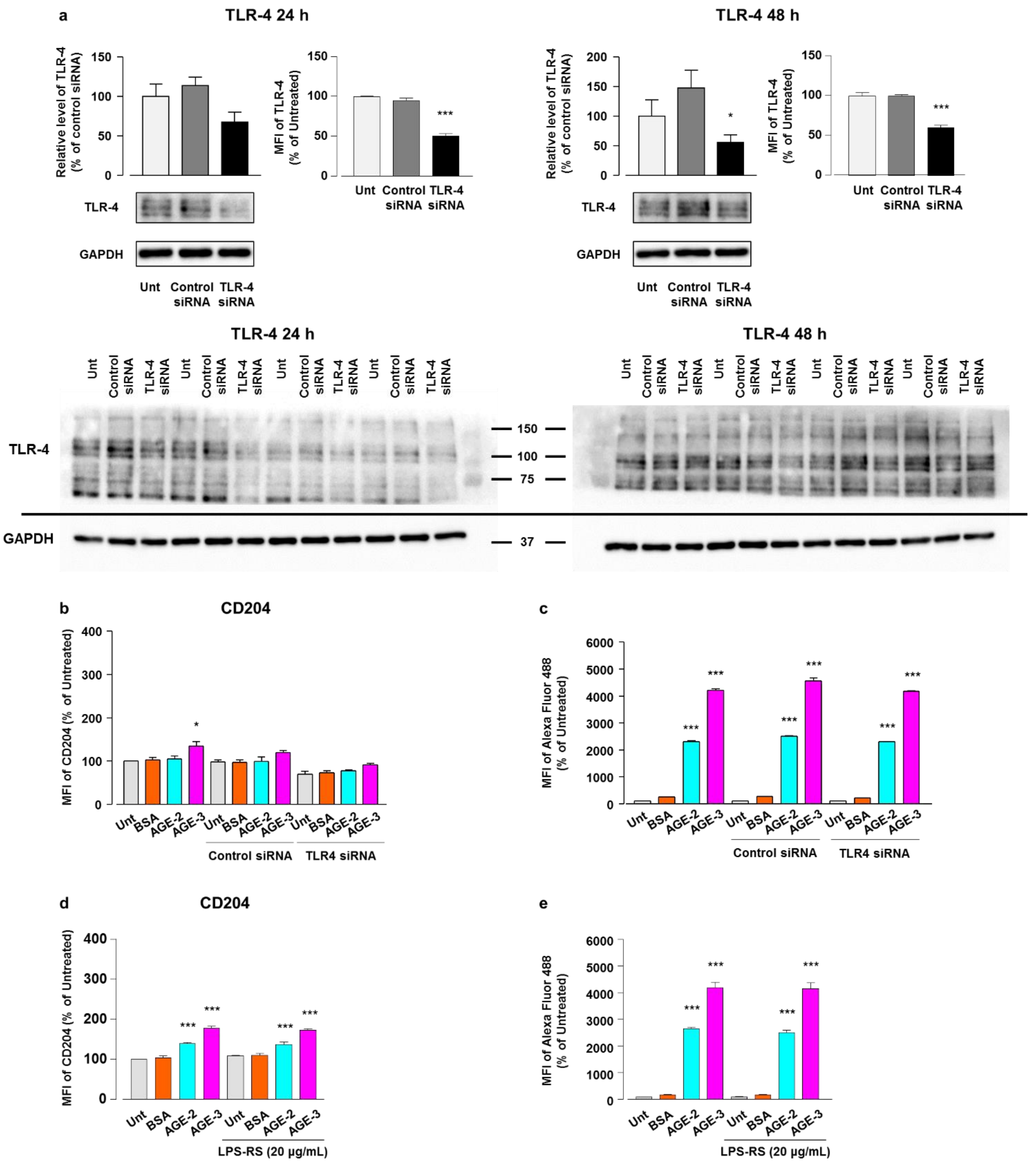

Supplementary Fig. S6

Supplementary Figure S7. AGE-2-and AGE-3-enhanced expression of CD204 is independent of NF-*κ*B activation. (a, b) The protein expression levels of NF-κB p65 and phospho (p)-NF-κB p65 were measured by western blotting using the whole cell lysates or nuclear extract of RAW264.7 cells exposed to AGEs (200 μg/ml) for 30 min at 37°C under 5% CO_2. Treatment of RAW264.7 cells with AGEs had little impact on the activation status of NF-κB signalling as determined by the ratio of the expression level of p-NF-κB/NF-κB in both cellular fractions. Relative level of p-NF-κB/NF-κB is shown as a ratio of (a) p-NF-κB/NF-κB relative to histone H3 in the nuclear extract and (b) p-NF-κB/NF-κB relative to GAPDH in the whole cellular fraction, normalized to the medium only group (Untreated cells; Unt). Data are expressed as the means ± SEM. (a) n = 15, (b) n = 7. (c) RAW264.7 cells were seeded at 2.0×10^5 per well in 24-well plates followed by pre-treatment for 1 h with or without the inhibitor of NF-*κ*B, PDTC at 20 μM or DMSO used as a vehicle of PDTC. Then, cells were further incubated with AGEs at 200 μg/ml for 1 h. PDTC had no effect on an increase in surface CD204 expression induced by AGE-2 and AGE-3 as determined by flow cytometry (n = 5). Each column represents the MFIs of CD204 relative to Unt, which was arbitrarily defined as 100%. Data are presented as the means ± SEM and analysed using one-way ANOVA followed by Tukey's test. $^{***}p < 0.001$ compared with the value for Unt.

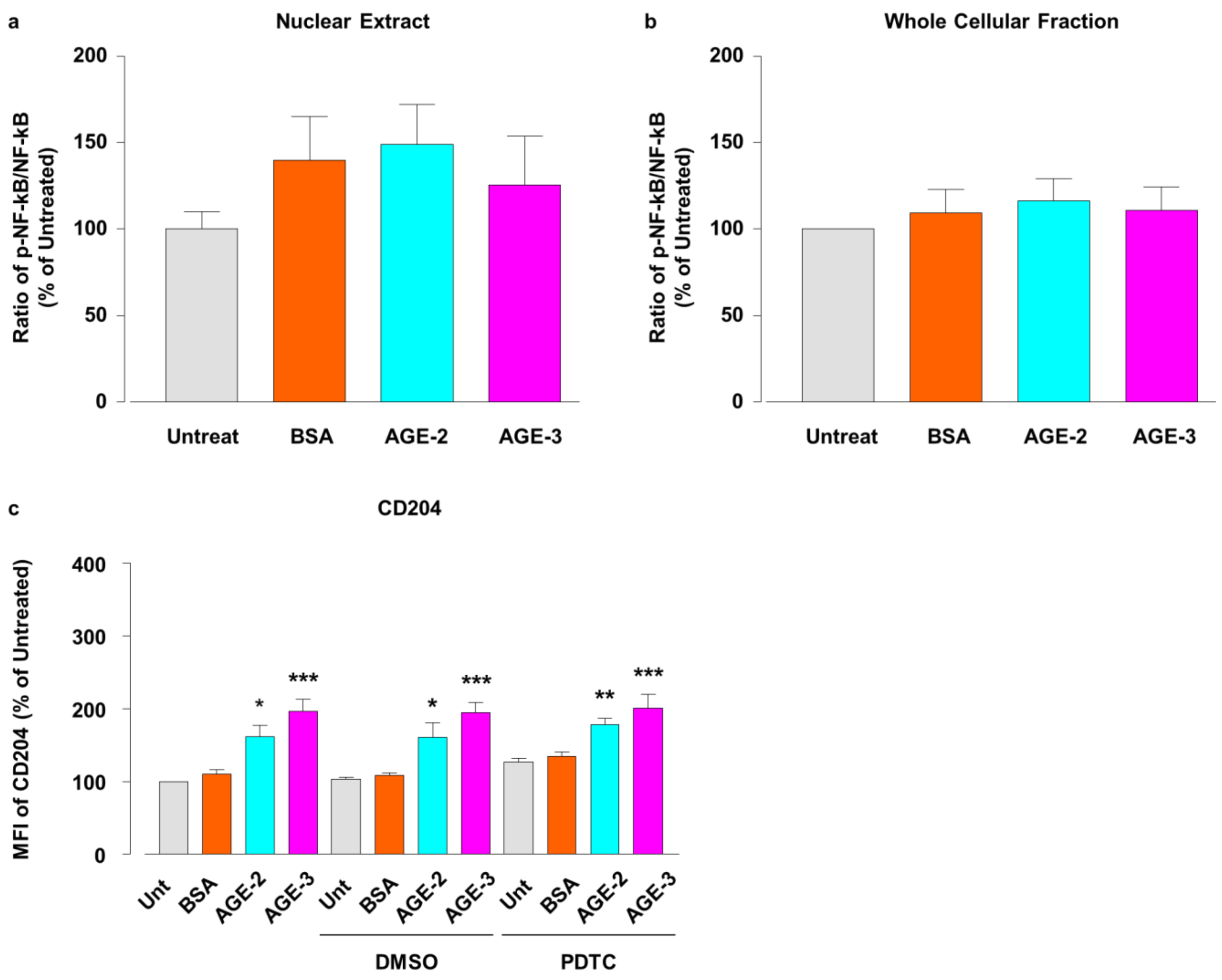

Supplementary Fig. S7

1

Supplementary Figure S8. Effect of a pinocytosis inhibitor on the AGEs uptake and the surface expression of CD204 in RAW264.7 cells. (a) After culturing RAW264.7 cells seeded at 1.0×10^5 per well for 1 h in the medium containing 5-(N-ethyl-N-isopropyl)-Amiloride (EIPA), a pinocytosis inhibitor, at the increasing concentrations ranged from 1–50 μM followed by treatment with Alexa Fluor 488-labelled AGEs at 200 μg/ml, the uptake levels of fluorescent AGEs in RAW264.7 cells were determined by flow cytometry analysis. Conversely, pre-treatment with EIPA dose-dependently but partially inhibited the uptake level of fluorescent AGE-2 and AGE-3 in RAW264.7 cells, implying that the fluid phase endocytosis (pinocytosis) is partly implicated in the uptake mechanism of AGEs by macrophage. n = 3. (b) After incubation of RAW264.7 cells seeded at 1.0×10^5 per well with EIPA at the concentrations ranged from 1–50 μM for 1 h, cells were exposed to AGEs at 200 μg/ml for 1 h. Subsequently, the surface expression level of CD204 in RAW264.7 cells was measured by flow cytometry analysis. Exposure to AGE-2 and AGE-3 both significantly increased CD204 expressions in comparison with medium only group (Untreated cell; Unt). Notably, pre-treatment with EIPA at any doses never had significant influences on increases in the CD204 expression level induced by AGE-2 and AGE-3. n = 3. All data are presented as the means ± SEM and analysed using one-way ANOVA followed by Tukey's test. $^{***}p < 0.001$, $^{*}p < 0.05$ compared with the value for Unt, $^{\#\#\#}p < 0.001$ compared with the value for AGE-2 alone, $^{\dagger\dagger\dagger}p < 0.001$, $^{\dagger}p < 0.05$ compared with the value for AGE-3 alone.

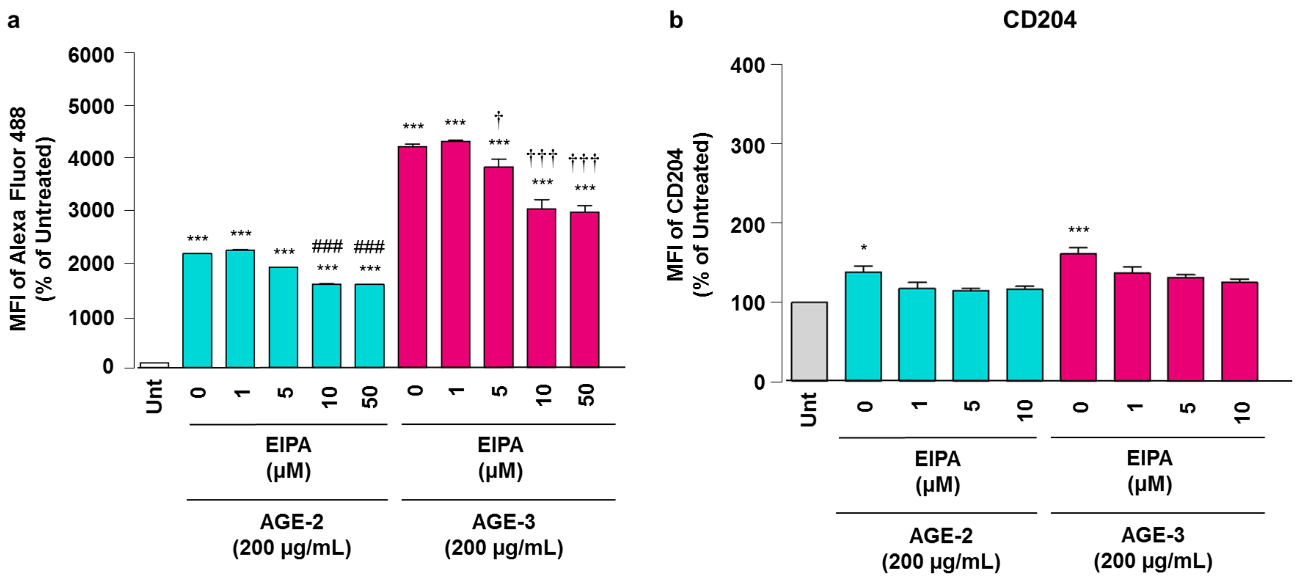

Supplementary Fig. S8

1

Supplementary Materials and Methods

Cell viability assay. RAW264.7 cells were seeded at 1.0 × 10^4 per well in 24-well plates and stimulated by non-labelled AGEs or Alexa Fluor 488-labelled-AGEs at the different concentrations ranging from 0.2 to 200 µg/ml. After incubating for 4 h at 37°C under 5% CO_2, cells were harvested and rinsed twice with FACS wash buffer consisting of phosphate-buffered saline (PBS) supplemented with 2.5% normal horse serum, 0.1% sodium azide, and 10 mM HEPES, followed by centrifugation (500 × *g*, 5 min, 4°C). Thereafter, the supernatant was removed, followed by addition of PBS. The remaining cells were stained with PI (2 µg/ml, Dojindo Laboratories) and analysed with a CantoII FACS (BD Biosciences, San Jose, CA, USA). Data were processed using FACSDiva software (BD Biosciences) to determine the percentage of PI-positive dead cells in each treatment group.

Time-lapse live-cell imaging during the endocytic uptake of AGEs. RAW264.7 cells were fluorescently labelled with PKH26 (red) (MINI26) according to the manufacturer's protocol. After attaching PKH26-labelled RAW264.7 cells seeded at 4.0 × 10^5 onto 35 mm glass bottom dishes for 1 h, cells were treated with 200 µg/ml Alexa Fluor 488-labelled -AGE2 or -AGE3. Next, the 35-mm dish was placed on the stage of an All-in-One fluorescence microscope BZ-X710 (Keyence, Osaka, Japan) equipped with an environmental chamber, which provided 37°C, humidity, and 5% CO_2 conditions. Time-lapse live-cell imaging was obtained at 0.5–1.0 µm intervals for the *z*-axis every 5 min over 4 h with a set of green and red emissions at original magnification ×400. Obtained images were processed and analysed using BZ-X710 software (Keyence).

Confocal laser scanning immunofluorescence microscopy. After adhering RAW264.7 cells seeded at 4.0 × 10^5 onto 35 mm glass bottom dishes for 1 h, cells were incubated in the presence of

AGEs (200 ng/ml) for 4 h at 37°C under 5% CO_2. Then, cells were washed with PBS (−) and fixed with 4% PFA at room temperature for 30 min followed by washing with PBS (−). Subsequently, cells were incubated in blocking buffer containing 1% BSA, 0.3 M glycine in PBS-T (PBS pH 7.6 with 0.1% Tween-20) at room temperature for 1 h to permeabilise the cell membrane and to block non-specific protein-protein interactions. Next, cells were incubated with normal rabbit IgG (1:400 for a negative control) or Ab directed against AGEs (1:400 dilution, ab23722, Abcam) in blocking buffer at 4°C overnight. After rinses in PBS-T, cells were incubated with Alexa Fluor 488-conjugated secondary Ab against rabbit IgG (1:500 dilution, A-11008, Thermo Fisher Scientific) at room temperature for 1 h. Then, cells were washed with PBS-T and photomicrographs were taken at 0.6–0.9 μm intervals for the *z*-axis at original magnification × 400 with a confocal laser C2 microscope (Nikon).

Flow cytometric analysis for macrophage pinocytosis of AGEs. RAW264.7 cells were seeded at 1.0×10^5 cells/well in 24-well plates, and subsequent incubation for 1 h with or without 5-(N-ethyl-N-isopropyl)-Amiloride (EIPA) (14406, Cayman Chemical, Ann Arbor, MI, USA) at the different concentrations ranging from 1 to 50 μM followed by treatment with Alexa Fluor 488-AGE-2 or AGE-3 at 200 μg/ml for 1 h. Thereafter, cells were harvested and processed twice by rinsing with FACS wash buffer consisting of PBS supplemented with 2.5% normal horse serum, 0.1% sodium azide, and 10 mM HEPES followed by centrifugation (200 × *g*, 5 min, 4°C). Subsequently, 300 μl PBS (−) was added to the residue and cells were stained with PI (2 μg/ml, Dojindo Laboratories) to exclude PI-

positive dead cells. After that, analysis was performed using FACS CantoII (BD Biosciences) and the data were processed using BD FACSDiva software (BD Biosciences) to determine the mean fluorescence intensity (MFI) of Alexa Fluor 488-labelled AGEs.

Flow cytometric analysis for CD204 expression in the surface membrane of RAW264.7 cells. RAW264.7 cells seeded at 1.0 × 10^5 per well in 24-well plates were incubated with Alexa Fluor 488-labelled AGEs (200 μg/ml) or non-labelled AGEs (200 μg/ml) for 1h. When conducting the experiments using pinocytosis inhibitor, RAW264.7 cells were incubated for 1 h in the presence or absence of EIPA (14406, Cayman Chemical) at the concentrations ranging from 1 to 50 μM followed by treatment with AGEs at 200 μg/mL for 1 h. Subsequently, cells were harvested and rinsed with FACS wash buffer followed by centrifugation (200 g, 5 min, 4°C), and then were stained with anti-mouse Ab against PE-conjugated CD204 (4 ng, 130-102-328, Miltenyi Biotec, Bergisch Gladbach, Germany) or its isotype matched control Ab (4 ng, 130-104-628, Miltenyi Biotec) at 4°C for 30 min. After rinsing with FACS wash buffer followed by centrifugation (200 g, 5 min, 4°C), 300 μl of PBS (-) was added to the residue followed by staining with PI (2 μg/ml) to exclude PI-positive dead cells. Thereafter, analysis was performed using a Canto II FACS and the data were processed using BD FACSDiva software to determine the MFI of PE.

Preparation for the whole cellular lysates of RAW264.7 cells. RAW264.7 cells seeded at 2.0 × 10^5 per well in 6-well plates were treated with BSA, AGE2, or AGE3 at 200 μg/ml for 30 min at 37°C under 5% CO_2. Then, cells were harvested, washed with PBS, and subsequently lysed in radio-immunoprecipitation assay buffer containing protease inhibitors for 30 min on ice. The supernatant of

the resulting suspension was obtained after centrifugation (16,000 × *g*, 30 min, 4°C) and collected as the total cell lysate. The total protein concentration was quantified using a Bradford protein assay kit.

Nuclear protein extraction from RAW264.7 cells. RAW264.7 cells seeded at 2.0 × 10^6 per dish in 100 mm i.d. dish were treated with AGEs (200 μg/ml) for 30 min at 37°C under 5% CO_2. Subsequently, cells were harvested and processed twice by washing with PBS followed by centrifugation (500 × *g*, 3 min, 4°C). After pellets were re-suspended in PBS and centrifuged (6,000 × *g*, 2 min, 4°C), supernatants of the resulting solution were removed. Subsequently, the procedure to obtain the nuclear extract fraction was performed using a Cytoplasmic & Nuclear Protein Extraction Kit (P504, 101Bio.com, Mountain View, CA, USA) according to the manufacturer's protocol.

Western blotting analysis. The whole cellular fraction and nuclear extract of RAW264.7 cells were diluted with an equal volume of 2 × sample buffer containing 0.5 M Tris-HCl (pH 6.8), 15% sodium dodecyl sulphate (SDS), 12% β-mercaptoethanol, 20% glycerol, and 0.1% bromophenol blue, then heated for 5 min at 97°C. Each protein fraction was subjected to SDS-polyacrylamide gel electrophoresis followed by electrotransfer onto a nitrocellulose membrane. The blotted membranes were blocked at room temperature for 1 h with blocking buffer containing 5% non-fat dry milk (Wako Pure Chemical Industries, Osaka, Japan) for GAPDH and histone H3, or 5% BSA (Wako Pure Chemical Industries) for NF-κB p65, phospho-NF-κB (p-NF-κB) p65, and TLR-4 in Tris-buffered saline (TBS)-T (TBS pH 7.6 with 0.1% Tween-20). The membrane was then probed with primary rabbit anti-mouse Abs against NF-κB p65 (8242, 1:2,000 dilution, Cell Signaling Technology Japan), p-NF-κB p65 (3033, 1:2,000 dilution, Cell Signaling Technology Japan), or TLR-4 (ab22048, 1:2,000 dilution, Abcam) as well as GAPDH (MAB374, 1:20,000 dilution, Merck Millipore, Darmstadt,

Germany) used as internal control for whole cellular fraction or histone H3 (4499, 1:2,000 dilution, Cell Signaling Technology Japan) used as internal control for nuclear extract in respective blocking buffer at 4°C overnight. Blots were then washed with TBS-T and incubated with horseradish peroxidase-conjugated anti-rabbit secondary Abs (074-1506, 1:4,000 dilution, Kirkegaad and Perry Laboratories, Guildford, UK) or anti-mouse secondary Abs (074-1806, 1:4,000 dilution, Kirkegaad and Perry Laboratories) in respective blocking buffer for 1 h at room temperature. After rinsing with TBS-T, the immune-complexes were visualized using Pierce ECL Western Blotting Substrate (Thermo Fisher Scientific). The intensities of immune reactive signals were measured using an Amersham Imager 600 CCD-based chemiluminescent analyser (GE Health Care Japan, Tokyo, Japan). For assessment of protein expression levels, the relative band intensity of each protein was estimated using Image Quant TL software (GE Healthcare Japan). Values for NF-κB p65, p-NF-κB p65, and TLR-4 relative to GAPDH or histone H3 were normalized to untreated cells in each fraction.

Treatment with TLR-4 siRNA in RAW264.7 cells. Stealth RNAi siRNA of TLR-4 and Stealth RNAi siRNA Negative Control, Lipofectamine RNAiMAX Reagent were purchased from Thermo Fisher Scientific. RAW264.7 cells were seeded in 24-well plates at 1.0×10^5 per well followed by treatment with the complex of siRNA (10 pmol/well) and Lipofectamine RNAiMAX Reagent for 24 h, after which cell culture medium was replaced by fresh medium. Subsequently, the knock-down cells were used for experiments in western blotting and flow cytometric analyses.
